# Supplementary material for: Bacterial motility can govern the dynamics of antibiotic resistance evolution
Source: Nat Commun. 2023 Sep 11;14:5584. doi: 10.1038/s41467-023-41196-8 (PMC10495427; doi:10.1038/s41467-023-41196-8)
Supplement: Supplementary file 1 — Supplementary Information [file 41467_2023_41196_MOESM1_ESM.pdf]

# Supplementary Information

## SUPPLEMENTARY NOTES

An important purpose of the Supplementary Notes is to present the analytical study of the formation of wild-type profiles in our models. In the main text, we claim that the wild-type profile is key for bacterial adaptation in an antibiotic gradient as it shapes the fitness landscape of mutants (see Results). The quantitative analysis of the wild-type profile provides the first step to the computation of the evolutionary time, the ecological time and the adaptation rate (see Methods). Therefore, understanding the formation of the wild-type profile is important for both intuitive and quantitative analysis.

The Supplementary Notes are organised into separate notes for convenience and clarity. We start by restating the analytical results for the wild-type profile of the simple source-sink model presented in the final subsection of Results. Starting from the end of the main text is deliberate because the source-sink model is a toy model, which provides an intuition for the formation of the wild-type profile in other more complex models considered in this work. However, each note within Supplementary Notes is relatively self-contained and the notes can be read in any order. We provide next a short guide to explain the content of each Supplementary Note and its links to other parts of our work:

- Supplementary Note 1: summary of results about the wild-type profile in the source-sink model presented in Fig. 5 (fourth subsection of Results).
- Supplementary Note 2: utilization of Supplementary Note 1 to explain why the bifurcation argument for the emergence of critical motilities applies to a large variety of models (fourth subsection of Results).
- Supplementary Note 3: wild-type profile in the staircase model presented in Fig. 1 and Fig. 2 (first and second subsections of Results).
- Supplementary Note 4: modified staircase models and their wild-type profile, including the effect of resistance costs in Supplementary Fig. 2, level of mutation rates in Supplementary Fig. 3, differential competition between growing and non-growing cells in Supplementary Fig. 4, bactericidal antibiotics in Supplementary Fig. 5, bacterial chemotaxis in Supplementary Fig. 6 (second subsection of Results) and horizontal gene transfer in Supplementary Fig. 9 (Discussion).
- Supplementary Note 5: wild-type profile in the model of stochastic phenotypic switching presented in Fig. 3 and Supplementary Fig. 7 (third subsection of Results).
- Supplementary Note 6: wild-type profile in the model of density-dependent motility presented in Fig. 4 and Supplementary Fig. 8 (third subsection of Results).
- Supplementary Note 7: explanation of how our theory links to particular experiments and explains the observed evolutionary dynamics (Discussion, Supplementary Table 1).
- Supplementary Note 8: supplementary methods detailing the computation of the survival probability of the first mutant in the overlap region.
- Supplementary Note 9: mathematical proofs of all theorems presented in all the Supplementary Notes mentioned above.
- Supplementary Note 10: supplementary methods detailing the exact parameter choices for each figure of our work, including information about main and supplementary figures.

### Supplementary Note 1. Source-sink model.

In the main text, we studied the wild-type profile of the source-sink model (staircase with $L = 2, R = 1$), described by the dynamical system with equation (4). The phase portraits of the system are shown in Fig. 5b and the results are summarised in Theorem 1.

**Theorem 1.** *System with equation* (4) *admits exactly two fixed points*

- *trivial fixed point:* $N_2 = N_1 = 0$,

- *non-trivial fixed point:*

$$N_1 = K\left(1 - \frac{\delta(\delta+2\nu)}{r(\delta+\nu)}\right),$$
$$N_2 = \frac{\nu}{\nu+\delta}N_1. \tag{S1}$$

*Exactly one of these fixed points is stable and corresponds to a stable wild-type profile, with non-negative cell numbers* $N_{1,2} \geq 0$. *Let* $\overline{f} = (r-2\delta)/2$ *be the average wild-type fitness. The stable wild-type profile corresponds to the non-trivial fixed point iff*

- *the environment is source-like* $\overline{f} > 0$ *and cell motility* $\nu$ *is arbitrary, or*
- *the environment is sink-like* $\overline{f} < 0$ *and cell motility is constrained to* $\nu \in [0, \nu_c]$*, where the critical motility is*

$$\nu_c = \frac{\delta(r-\delta)}{2\delta - r} > 0. \tag{S2}$$

There are two implications of Theorem 1. First, this theorem provides an explanation for the existence of a critical motility as explained in the fourth subsection of Results and further discussed in the next Supplementary Note 2. Second, it explains the shape of the wild-type profile $N_x$ in Fig. 1d. The population in compartment $x = 2$ is significantly suppressed at low motility ($\nu \ll \delta$) and the population is homogeneously distributed across space at high motility ($\nu \gg \delta$). We elaborate on this phenomenon in the Supplementary Note 3.

**Supplementary Note 2. Robust mechanism for the emergence of the critical motility.**

In the main text, we claimed that the critical motility can be understood as a bifurcation point of a dynamical system for the wild-type profile formation and that this mechanism is applicable to a large variety of models. Below we first provide a heuristic argument for why critical motilities shall arise for all models in our paper, and then we make a comparison with critical motilities in ecological models.[S1–S3]

Theorems with analogous structure to Theorem 1 shall exist for more complicated models of our paper and shall predict the existence of a critical motility. Heuristically, we can argue as follows. Vary motility $\nu \geq 0$ as the bifurcation parameter. When there is no motility $\nu = 0$, the dynamics between spatial compartments decouples and the non-trivial fixed point is stable while the trivial fixed point is not. As cell motility increases, the non-negative quadrant $N_x \geq 0$ becomes forward invariant and its boundary contains a single fixed point, the trivial fixed point. Assuming that the underlying Markov process is ergodic, a unique stable fixed point must exist within the non-negative quadrant $N_x \geq 0$. This implies that the non-trivial fixed point either stays in the positive quadrant and remains stable, or it leaves the positive quadrant through a transcritical bifurcation with the trivial fixed point, see Fig. 5b. Populations of high motility are generically governed by the average fitness,[S4] explaining the stability of the trivial fixed point at high motility in sink-like environments. Therefore, there must exist a critical motility associated with the transcritical bifurcation if the environment is sink-like.

In the main text, we claimed that this bifurcation mechanism also applies to the critical motility in ecological models that study range expansion.[S1–S3] We elaborate on this claim with a migration-selection model from[S3] and relate this model to our source-sink model. Consider two alleles $a$ and $A$ and two habitats, such that allele $A$ is selected for in habitat 1 with strength $s$ and allele $a$ is selected for in habitat 2 with strength $\alpha s$ ($\alpha, s > 0$). Moreover, these alleles can move between habitats at rate $\nu$. The relative frequencies of allele $a$ in habitat $i \in \{1, 2\}$ are denoted by $a_i \in [0, 1]$, and their dynamics can be described by a replicator equation with constant selection:

$$\dot{a}_1 = s a_1(1-a_1) - \nu a_1 + \nu a_2$$
$$\dot{a}_2 = -\alpha s a_2(1-a_2) + \nu a_1 - \nu a_2, \tag{S3}$$

This is a dynamical system on a square $(a_1, a_2) \in [0, 1]^2$. When $\nu = 0$, there is a globally attracting non-trivial fixed point $(a_1, a_2) = (1, 0)$. When $\nu > 0$, the square $[0, 1]^2$ becomes forward invariant and the boundary supports two trivial fixed points: $(a_1, a_2) = (0, 0)$ (allele $A$ wins) and $(a_1, a_2) = (1, 1)$ (allele $a$ wins). Moreover, as $\nu$ increases from 0, the globally attracting fixed point is pushed from the boundary of the square into the interior. Similarly to the source-sink model, the non-trivial fixed point can leave the square only through a transcritical bifurcation with one of the trivial fixed points, giving rise to a critical motility $\nu_c$. A straightforward calculation for the non-trivial fixed

point shows that it collides with the trivial fixed point $(a_1, a_2) = (0, 0)$ when $\alpha > 1$ and with the trivial fixed point $(a_1, a_2) = (1, 1)$ when $0 < \alpha < 1$ at

$$\nu_c = \frac{s\alpha}{|1-\alpha|}. \tag{S4}$$

Therefore, in accordance with,[S3] both alleles coexist if $\nu < \nu_c$ and the overall more fit allele takes over if $\nu > \nu_c$.

The similarity between this model and the source-sink model is not a coincidence. The source-sink system with equation (4) can be manipulated and rearranged into

$$\begin{aligned} \dot{a_1} &= (r-\delta)a_1(1-a_1) - \nu a_1 + \nu a_2 - \delta a_1^2, \\ \dot{a_2} &= -\delta a_2(1-a_2) + \nu a_1 - \nu a_2 - \delta a_2^2, \end{aligned} \tag{S5}$$

by relabelling $N_i \equiv a_i \in [0, \infty)$. This is similar to equation (S3) with $s = r - \delta$, $\alpha = \delta/(r-\delta)$ near the trivial fixed point $(a_1, a_2) = (0, 0)$. However, the point $(a_1, a_2) = (1, 1)$ is no longer a fixed point due to the corrections of $-\delta a_i^2$. Therefore, when $\alpha > 1$, the environment is sink-like and a critical motility exists, with equation (S4) matching equation (S2) upon substitution of $s$ and $\alpha$. When $0 < \alpha < 1$, the environment is source-like and no critical motility exists, since the corresponding trivial fixed point is removed from the dynamics by the correction terms $-\delta a_i^2$.

## Supplementary Note 3. Staircase model.

In the main text, we studied the shape of the wild-type profile in the staircase model and its impact on mutant fitness (Fig. 1d). Hereby, we study this process analytically by using the same framework as for the source-sink model (Supplementary Note 1, fourth subsection of Results). The wild-type profile $N_x$ at state $R$ of the adaptation process (Fig. 1b) is governed by a dynamical system with equation (S6) on $\mathbb{R}^L$, where $N_x$ is the total number of cells in spatial compartment $x$:

$$\begin{aligned} \dot{N_1} &= r\left(1 - \frac{N_1}{K}\right)N_1 && + \nu N_2 && -(\nu+\delta)N_1, \\ \dot{N_x} &= r\left(1 - \frac{N_x}{K}\right)N_x && +\nu N_{x-1} + \nu N_{x+1} && -(2\nu+\delta)N_x, \; x = 2, \ldots, R \\ \dot{N_x} &= && \nu N_{x-1} + \nu N_{x+1} && -(2\nu+\delta)N_x, \; x = R+1, \ldots, L-1 \\ \dot{N_L} &= && \nu N_{L-1} && -(\nu+\delta)N_L., \end{aligned} \tag{S6}$$

Similarly to the source-sink model, the non-negative quadrant $N_x \geq 0$ can only contain the trivial and the non-trivial fixed point[?] . Let us concentrate on the non-trivial fixed point $N_x$. While a closed form expression for the fixed point does not exist for $R > 1$, important properties of the non-trivial fixed point $N_x$ can be derived analytically. To study the curvature of the wild-type profile, we define the convexity of the profile[?] .

**Definition 1.** *$N_x$ is convex, resp. concave on $[a, b]$ if for any $x \in [a, b] \cap \{1, \ldots, L\}$:*

$$\frac{N_{x+1} + N_{x-1}}{2} > N_x, \; \textit{resp.} \; \frac{N_{x+1} + N_{x-1}}{2} < N_x.$$

Using this definition, important qualitative properties of the wild-type profile can be derived.

**Theorem 2.** *Let $N_x$ be any fixed point of the dynamical system with equation* (S6) *that lies in the strictly positive quadrant $N_x > 0$. Note that $N_x$ coincides with the non-trivial fixed point $N_x$ if it is stable. This wild-type profile $N_x$ of resistance $R$ satisfies:*

1. *$N_x$ is a decreasing function of $x$.*
2. *In any compartment above the staircase, the effective wild-type birth rate $r(1 - N_x/K)$ is bigger than the death rate $\delta$.*
3. *$N_x$ is concave above the staircase, i.e., on $[1, R+1]$.*
4. *$N_x$ is convex below the staircase, i.e., on $[R, L]$.*

5. *The curvature of the wild-type population profile at position $x$ is proportional to wild-type fitness $r(1 - N_x/K)N_x\mathbb{1}_{x\leq R} - \delta N_x$ at this position and inversely proportional to motility.*

6. *The mutant fitness $r(1 - N_x/K)N_x\mathbb{1}_{x\leq R+1} - \delta N_x$ is a positive, convex and increasing function on $[1, R+1]$, and a constant $-\delta$ function on $[R+2, L]$. In particular, mutant fitness is maximised in the overlap region and matches the wild-type fitness characterised above in compartments $x \leq R$.*

7. *In any compartment $N_x < K(1 - \delta/r)$.*

8. *$r > \delta$ is a necessary condition for the existence of this non-trivial fixed point in the strictly positive quadrant.*

Theorem 2 explains the generic shape of a wild-type profile for any motility $\nu$ (Fig. 1d) and the shape of the mutant fitness landscape. However, it does not explain the difference between the low and high motility regimes discussed in the main text. In order to understand the behaviour of the wild-type profile at low and high motility, asymptotic expansion for the non-trivial fixed point are needed.

**Theorem 3.** *In the low motility regime $\nu \ll \delta$, the non-trivial fixed point is given by:*

$$N_x = \begin{cases} K\left(1 - \frac{\delta}{r}\right) + O\left(\left(\frac{\nu}{\delta}\right)^2\right) & \text{if } x = 1, \dots, R-1 \\ K\left(1 - \frac{\delta+\nu}{r}\right) + O\left(\left(\frac{\nu}{\delta}\right)^2\right) & \text{if } x = R \\ K\frac{\nu}{\delta}\left(1 - \frac{\delta}{r}\right) + O\left(\left(\frac{\nu}{\delta}\right)^2\right) & \text{if } x = R+1 \\ 0 + O\left(\left(\frac{\nu}{\delta}\right)^2\right) & \text{if } x = R+2, \dots, L \end{cases} \tag{S7}$$

*In the high motility regime $\nu \gg \delta$, the non-trivial fixed point is given by:*

$$N_x = K\left(1 - \frac{\delta L}{rR}\right) + b_x\frac{\delta}{\nu} + O\left(\left(\frac{\delta}{\nu}\right)^2\right), \tag{S8}$$

*where the coefficients $b_x$ satisfy*

$$\begin{aligned} b_1 &= \frac{\delta K}{6rR}\left(3L^3 - 3L^2R + 3LR^2 + (R-L)(R^2-1)\left(\frac{2L}{R} - \frac{r}{\delta}\right)\right), \\ b_x &= b_1 + K\left(1 - \frac{\delta L}{rR}\right)\frac{x(x-1)}{2}\left(1 - \frac{L}{R}\right), \text{ if } x = 2, \dots, R+1, \\ b_x &= b_1 + K\left(1 - \frac{\delta L}{rR}\right)\left(\frac{x(x-1) + L(1+R-2x)}{2}\right), \text{ if } x = R, \dots, L. \end{aligned} \tag{S9}$$

Using Theorem 3, we can see that the shape of the population front is always the same in the low motility regime and overlap region $x = R+1$ provides a refuge for mutants (Fig. 1d). The high motility regime leads to a homogeneous wild-type population $N_x$, which increases with resistance state $R$ and competes with mutants for space (Fig. 1d). Moreover, this fixed point can be in the negative quadrant to first order if the environment is sink-like. In this case, the non-trivial fixed point leaves the positive quadrant $N_x > 0$ at a critical motility $\nu_c$ and exchanges its stability with the trivial fixed point via a transcritical bifurcation.

**Corollary 1.** *Let $\overline{f} = (rR - \delta L)/L$ be the average wild-type fitness. Then, the stable wild-type profile is non-vanishing iff*

- *the environment is source-like $\overline{f} > 0$ and motility $\nu$ is arbitrary, or*
- *the environment is sink-like $\overline{f} < 0$ and motility is constrained to $\nu \in [0, \nu_c]$, where to first order*

$$\nu_c = \frac{\delta b_L}{K\left(\frac{\delta L}{rR} - 1\right)}. \tag{S10}$$

In the main text, we find a deadly motility regime characterised by extinction and no adaptation when $\nu > \nu_c$. We can prove that the adaptation rate is vanishing by deriving its upper bound.

**Theorem 4.** *Fix a resistance state $R$ and define $a_{evo} = 1/\mathbb{E}T^R_{evo}$. Then,*

$$a_R < a_{evo} < \mu_f \sum_x N_x,$$

*In particular, when critical motility $\nu_c$ exists, the adaptation rate $a_R$ decreases to 0 faster than $\mu_f \sum_x N_x$ as $\nu \to \nu_c^-$. Moreover, no adaptation can occur if the wild-type population is extinct.*

## Supplementary Note 4. Modified staircase model.

In the main text, we noted that the staircase model can be modified to consider the effect of fitness costs of resistance (Supplementary Fig. 2), level of mutation rates (Supplementary Fig. 3), differential competition between growing and non-growing cells (Supplementary Fig. 4), bactericidal antibiotics (Supplementary Fig. 5), bacterial chemotaxis (Supplementary Fig. 6) and horizontal gene transfer (Supplementary Fig. 9). In this Supplementary Note, we explain each of these modifications of the original staircase model in turn and present the results for the modified models, with the key conclusion that motility governs the bacterial adaptation by different mechanisms in the low motility, high motility and deadly motility regimes as identified in the original staircase model (Fig. 2a). We finish this Supplementary Note with a mathematical analysis of the wild-type profile.

*a. Resistance costs.* The original staircase model assumes that there are no fitness costs associated to resistance. Yet, adaptations that confer antibiotic resistance often have a cost that is expressed in the absence of antibiotic pressure, and in these cases resistant genotypes often grow slower than susceptible ones.[S5,S6] We model such fitness costs of resistance by decreasing the division rate of cells above the staircase $g \geq x$ by a factor of $(1-c)^{g-x}$, where $c \in [0,1)$ is the resistance cost.[S7] We find that sufficiently high resistance costs can decrease the adaptation rate $a_R$ when cell motility is sufficiently low (Supplementary Fig. 2). This agrees with Hermsen *et al.*[S8] that shows that the resistance cost $c$ affects the adaptation rate precisely when cell motility is very low ($\nu < c^2\delta$). The rationale for this result is simple. When cell motility is low, resistant genotypes that harbour a resistance cost are less able to avoid competition with the wild-type and cannot easily move to where they have higher growth advantage. Importantly, we find that resistance costs do not affect our key conclusion: low motility accelerates adaptation, high motility decelerates adaptation and very high motility leads to extinction if the average wild-type fitness $\overline{f} = rR/L - \delta$ is negative. As a result, the deadly motility regime is not influenced by resistance costs (Supplementary Fig. 2).

*b. Level of mutation rates.* In the original staircase model and all its modifications, we assume that mutation rate is small compared to the other rates. This is an important assumption that allows us to perform the mathematical analysis of wild-type profiles as presented throughout the Supplementary Notes. To test the limits of this assumption, we also consider higher mutation rates. Specifically, we vary the the forward (resistance-conferring) mutation rate $\mu_f$ (Supplementary Fig. 3). We find that mutation rates below $\mu_f < 10^{-4}$ do not affect the relationship between adaptation rate and motility: adaptation rate increases in the low motility regime, decreases in the high motility regime, and no adaptation occurs in the deadly motility regime. This mutation rate threshold corresponds to the probability of $\mu_f/\delta = 10^{-3}$ resistance mutations per cell division, which is very high compared to experimental estimates of $10^{-6}$ and $10^{-9}$ resistance mutations per cell division.[S9–S11]

*c. Competitive strength of non-growing cells.* In our models, we assume logistic growth and non-growing cells can compete with growing cells by contributing to the carrying capacity via influx from other compartments. For simplicity, we started by assuming that growing and non-growing cells compete with each other with the same strength, but we also considered the effect of reduced competitive strength of non-growing cells on our conclusions. The reduced competitiveness of non-growing cells is modelled by $\alpha \in [0,1]$ which corresponds to the proportion of the non-growing cells that contribute to the carrying capacity. In particular, $\alpha = 0$ corresponds to no competitiveness of the non-growing cells and $\alpha = 1$ corresponds to the same competitiveness of growing and non-growing cells. More specifically, the division rate of cells growing above the staircase ($g \geq x$) is modified from $\max(0, r(1 - N_x/K))$ to $\max(0, r(1 - (N_x^g + \alpha N_x^n)/K)$, where $N_x^g$ is the number of growing cells above the staircase ($g \geq x$) and $N_x^n$ is the number of non-growing cells below the staircase ($g < x$) in a given spatial compartment $x$. We note that the relationship between adaptation rate $a_R$ and motility rate $\nu$ remains the same (Supplementary Fig. 4): the adaptation rate increases with low motility ($\nu < \delta$), decreases with high motility ($\nu > \delta$) and very high motility ($\nu > \nu_c$) leads to extinction if the average wild-type fitness $\overline{f} = rR/L - \delta$ is negative. The analytical theory suggests that the competitiveness $\alpha$ does not influence the wild-type profile and the evolutionary time $T_{\text{evo}}^R$. The competitiveness $\alpha$ can only modify the ecological time $T_{\text{eco}}^R$, which affects the evolutionary dynamics only if motility is high (Fig. 2c). In contrast, the low motility regime is not affected by $\alpha$, as it is dominated by the evolutionary time $T_{\text{evo}}^R$ that is independent of $\alpha$, and the deadly motility regime is not affected by $\alpha$, as it is determined by the wild-type profile that is also independent of $\alpha$.

*d. Bactericidal antibiotics.* The original staircase model studies the effect of antibiotics that inhibit bacterial growth (i.e., we considered bacteriostatic antibiotics). However, many natural and clinical antibiotics kill bacteria (bactericidal antibiotics). We model bactericidal antibiotics by increasing the death rate under the staircase $\sigma$-times as in Hermsen *et al.*.[S7] Importantly, our results show that bacterial adaptation to bactericidal antibiotics again has the same regimes dictated by cell motility (Supplementary Fig. 5). Interestingly, while bacterial adaptation is identical to the one found for bacteriostatic antibiotics in the low motility regime, a deadly motility regime can be induced at high motility if the bactericidal effect is sufficiently strong $\sigma \gg 1$. In this case, bacteria experience deadly antibiotic concentrations more often and the average wild-type fitness $\overline{f}$ is decreased by $\sigma$.

*e. Bacterial chemotaxis.* The original staircase model assumes that bacteria move randomly. Yet, it is well-known that bacteria can bias their motion in chemical gradients (chemotaxis). In particular, it has been shown that antibiotics can trigger both positive[S12] and negative[S13] chemotaxis. We model the effect of biased motility by changing the probability $p$ of bacterial movement up or down the antibiotic gradient. Notably, the usual adaptation regimes can be identified in this model (Supplementary Fig. 6). Our results show that chemotaxis has little impact on the adaptation rate when cell motility is low (Supplementary Fig. 6), mimicking what we found for bactericidal antibiotics described above. However, when motility is high, positive chemotaxis $p > 0.5$ increases antibiotic exposure and promotes the deadly motility regime, while negative chemotaxis $p < 0.5$ decreases antibiotic exposure and prevents the deadly motility regime (Supplementary Movie 2). Put differently, chemotaxis changes the average wild-type fitness $\overline{f}$, decreasing it for positive chemotaxis and increasing it for negative chemotaxis. For negative chemotaxis, the wild-type has higher average fitness but resides far from higher antibiotic concentrations. Mutants are thus less likely to reach the overlap region and the adaptation rate is decreased (Supplementary Fig. 6).

*f. Horizontal gene transfer.* Bacteria can exchange resistance genes through horizontal gene transfer (HGT), which is known to accelerate bacterial adaptation.[S14] In particular, it has been shown that the immigration of susceptible cells from a detached habitat can promote adaptation in a focal habitat via HGT.[S15] However, for closed systems without immigration of susceptible genotypes, it has been argued that HGT does not affect bacterial adaptation substantially.[S16–S20] In an attempt to recapitulate these results, we model HGT in our system by allowing less resistant genotypes $g$ to acquire resistance from $N_{x,g'}$ more resistant cells of genotype $g' > g$ located at the same position $x$ at a rate $h \times N_{x,g'}$. For realistic values of HGT rate $h$ ($< 1$ transfer per generation[S15]), we find that the adaptation rate is slightly increased, but our key conclusion still holds: motility accelerates bacterial adaptation in the low motility regime, while it decelerates bacterial adaptation in the high motility regime. Moreover, we find that bacteria with very high motility experience deadly motility regimes if they have negative average fitness $\overline{f} = rR/L - \delta$, irrespective of HGT rate (Supplementary Fig. 9).

*g. Wild-type profile.* So far, we considered various modifications of the staircase model. We now aim to understand how the conditions for the deadly motility regime change in the modified models. We see that there is no change to the deadly motility regime in the case of resistance costs, elevated mutation rates, differential competition between growing and non-growing cells, and HGT (Supplementary Fig. 2, 3, 4, 9). Therefore, we focus on the bactericidal antibiotics and bacterial chemotaxis that impact the deadly motility regime by modulating the average wild-type fitness $\overline{f}$ (Supplementary Fig. 5, 6). We find the formulae for the average wild-type fitness $\overline{f}$ in these two modified models by studying the wild-type profiles in general modified staircase models with spatially variable division rate $r_x$, death rate $\delta_x$ and motility rate $\nu_x$. In this setting, chemotaxis corresponds to the choice $\nu_{R,x} = 2p\nu$ and $\nu_{L,x} = 2(1-p)\nu$, where $p$ is the probability to move up the gradient; the effect of bactericidal antibiotics corresponds to stress-induced death rate $\delta_x = \sigma\delta$ below the staircase ($x > R$) and the usual death rate $\delta_x = \delta$ above the staircase ($x \le R$). The wild-type profile formation at state $R$ of the adaptation process (Fig. 1b) is governed by the mean-field equations:

$$\begin{aligned}
\dot{N}_1 &= r_1\left(1-\frac{N_1}{K}\right)N_1 && + \nu_{L,1}N_2 && -(\nu_{R,1}+\delta_x)N_1, && \\
\dot{N}_x &= r_x\left(1-\frac{N_x}{K}\right)N_x && +\nu_{R,x-1}N_{x-1}+\nu_{L,x}N_{x+1} && -(\nu_{R,x}+\nu_{L,x-1}+\delta_x)N_x,\ x && = 2,\dots,L-1 \qquad \text{(S11)}\\
\dot{N}_L &= r_L\left(1-\frac{N_L}{K}\right)N_L && +\nu_{R,L-1}N_{L-1} && -(\nu_{L,L-1}+\delta_L)N_L. &&
\end{aligned}$$

This dynamical system has two fixed points of interest, the trivial fixed point $N_x = 0$ and the non-trivial fixed point, which correspond to possible wild-type populations. Next, we compute the non-trivial fixed point at low and high motility and derive results for the existence of a critical motility. To do this, we assume that $\nu_{L,x}, \nu_{R,x} \sim O(\nu)$, $\delta_x \sim O(\delta)$, which is true for the modifications mentioned above.

**Theorem 5.** *In the low motility regime $\nu \ll \delta$, the wild-type profile is given by:*

$$N_x = \begin{cases} K\left(1-\frac{\delta_x}{r_x}\right) + K\left(\frac{r_{x+1}-\delta_{x+1}}{r_x-\delta_x}\frac{\nu_{L,x}}{r_{x+1}} - \frac{\nu_{R,x}}{r_x}\right) + O\left(\left(\frac{\nu}{\delta}\right)^2\right) & \text{if } x = 1\\ K\left(1-\frac{\delta_x}{r_x}\right) + K\left(\frac{r_{x+1}-\delta_{x+1}}{r_x-\delta_x}\frac{\nu_{L,x}}{r_{x+1}} + \frac{r_{x-1}-\delta_{x-1}}{r_x-\delta_x}\frac{\nu_{R,x-1}}{r_{x-1}} - \frac{\nu_{R,x}+\nu_{L,x-1}}{r_x}\right) + O\left(\left(\frac{\nu}{\delta}\right)^2\right) & \text{if } x = 2,\dots,L-1\\ K\left(1-\frac{\delta_x}{r_x}\right) + K\left(\frac{r_{x-1}-\delta_{x-1}}{r_x-\delta_x}\frac{\nu_{R,x-1}}{r_{x-1}} - \frac{\nu_{L,x-1}}{r_x}\right) + O\left(\left(\frac{\nu}{\delta}\right)^2\right) & \text{if } x = L \end{cases} \qquad \text{(S12)}$$

*In the high motility regime $\nu \gg \delta$, the wild-type profile is given by:*

$$N_x = \left[\prod_{y=1}^{x-1}\frac{\nu_{R,y}}{\nu_{L,y}}\right] K \left[\frac{\sum_{z=1}^{L}(r_z-\delta_z)\prod_{y=1}^{z-1}\frac{\nu_{R,y}}{\nu_{L,y}}}{\sum_{z=1}^{L} r_z \prod_{y=1}^{z-1}\frac{\nu_{R,y}}{\nu_{L,y}}}\right] + O\left(\frac{\delta}{\nu}\right). \qquad \text{(S13)}$$

*The average wild-type fitness therefore is* $\overline{f} = \sum_{z=1}^{L}(r_z - \delta_z)\prod_{y=1}^{z-1}\frac{\nu_{R,y}}{\nu_{L,y}} / \sum_{z=1}^{L}\prod_{y=1}^{z-1}\frac{\nu_{R,y}}{\nu_{L,y}}$. *The stable non-vanishing wild-type profile exists iff*

1. *the environment is source-like* $\overline{f} > 0$ *and motility* $\nu$ *is arbitrary, or*
2. *the environment is sink-like* $\overline{f} < 0$ *and motility is constrained to* $\nu \in [0, \nu_c]$ *for a critical motility* $\nu_c$.

We note that this result coincides with the one from the basic staircase model (SI Theorem 3, SI Corollary 1) if $r_x = r$ above the staircase and 0 otherwise, $\delta_x = \delta$ and $\nu_{R,x} = \nu_{L,x} = \nu$. Furthermore, this result explains the forms of the average wild-type fitness $\overline{f}$ that were presented above for bactericidal antibiotics and chemotaxis.

For bactericidal antibiotics, the antibiotics-induced death increases $\sigma$-times, which decreases the average wild-type fitness $\overline{f}$ below zero if $\sigma$ is sufficiently high, as

$$\overline{f} = (r-\delta)\frac{R}{L} - \sigma\delta\frac{L-R}{L}. \tag{S14}$$

Therefore, Theorem 5 explains why the bactericidal effect $\sigma$ converts the high motility regime into the deadly motility regime, while leaving adaptation in the low motility regime almost unchanged (Supplementary Fig. 5). Intuitively, at low motility regime the population is not present in stressful regions below the staircase, making the adaptation invariant of antibiotics-induced death. In the high motility regime, the population experiences the average environment with increased overall death rate, which decreases the average wild-type fitness.

For chemotaxis, probability $p$ that a cell moves up the antibiotic gradient decreases (resp. increases) the average wild-type fitness $\overline{f}$ below (resp. above) zero when $p$ is sufficiently high (resp. low), as

$$\overline{f} = r(1-p)^{L-R}\frac{p^R + p^{R-1}(1-p) + \cdots + p(1-p)^{R-1} + (1-p)^R}{p^L + p^{L-1}(1-p) + \cdots + p(1-p)^{L-1} + (1-p)^L} - \delta. \tag{S15}$$

This result means that positive chemotaxis can always create a deadly motility regime (irrespective of $R$), and negative chemotaxis can always prevent a deadly motility regime (irrespective of $R$), as we confirm with simulations (Supplementary Fig. 6). Theorem 5 also shows that, at high motility, the wild-type profile of a population with negative chemotaxis becomes concentrated away from the overlap region. This result explains the reduction in the adaptation rate in Supplementary Fig. 6 as a consequence of reduced antibiotic exposure. Moreover, Theorem 5 also predicts that chemotaxis does not affect the wild-type at low motility, explaining why the adaptation rate is not affected at low motility in Supplementary Fig. 6. We note that it is easier to introduce the deadly motility regime by controlling chemotaxis (power law dependence on $p$) rather than controlling bactericidal effects of antibiotics (linear dependence on $\sigma$).

## Supplementary Note 5. Stochastic switching of motility phenotypes.

In the main text, we studied the wild-type profiles of phenotypically heterogeneous populations with bacteria switching between different motility rates stochastically. Hereby, we study this process analytically. For convenience, we modify the notation slightly. The phenotypes have cell numbers $N_x$ and $M_x$; motilities $\nu_N$ and $\nu_M$; and phenotypes switch stochastically at rate $s$. Without loss of generality, we assume that $\nu_M \geq \nu_N$. At ecological timescales, the wild-type profile is governed by a dynamical system with equation (S16):

$$\begin{aligned}
\dot{N}_1 &= r\left(1 - \frac{N_1+M_1}{K}\right)N_1 + \nu_N N_2 + sM_1 - (\nu_N+\delta+s)N_1,\\
\dot{N}_x &= r\left(1 - \frac{N_x+M_x}{K}\right)N_x + \nu_N N_{x-1} + \nu_N N_{x+1} + sM_x - (2\nu_N+\delta+s)N_x,\ x=2,\ldots,R\\
\dot{N}_x &= +\nu_N N_{x-1} + \nu_N N_{x+1} + sM_x - (2\nu_N+\delta+s)N_x,\ x=R+1,\ldots,L-1\\
\dot{N}_L &= +\nu_N N_{L-1} + sM_L - (\nu_N+\delta+s)N_L,\\
\dot{M}_1 &= r\left(1 - \frac{N_1+M_1}{K}\right)M_1 + \nu_M M_2 + sN_1 - (\nu_M+\delta+s)M_1,\\
\dot{M}_x &= r\left(1 - \frac{N_x+M_x}{K}\right)M_x + \nu_M M_{x-1} + \nu_M M_{x+1} + sN_x - (2\nu_M+\delta+s)M_x,\ x=2,\ldots,R\\
\dot{M}_x &= +\nu_M M_{x-1} + \nu_M M_{x+1} + sN_x - (2\nu_M+\delta+s)M_x,\ x=R+1,\ldots,L-1\\
\dot{M}_L &= +\nu_M M_{L-1} + sN_L - (\nu_M+\delta+s)M_L,
\end{aligned} \tag{S16}$$

As before, the wild-type profile approaches the unique stable fixed point in the non-negative quadrant, which is either the trivial or the non-trivial fixed point. We next discuss the dynamics in the source-sink model ($L = 2, R = 1$) and state the results for the staircase model (any $L, R$).

In the source-sink model, there is a closed form expression for the wild-type profile.

**Theorem 6.** *The dynamical system with equation* (S16) *for the source-sink model (*$L = 2, R = 1$*) has two fixed points:*

- *the trivial fixed point:* $N_{1,2} = M_{1,2} = 0$,
- *the non-trivial fixed point:*

$$\begin{aligned} N_1 &= \frac{K}{1+B(s)}\left(1 - \frac{c_N(s) - d(s)B(s)}{r}\right), \\ M_1 &= \frac{K}{1+B(s)^{-1}}\left(1 - \frac{c_M(s) - d(s)B(s)^{-1}}{r}\right), \\ N_2 &= a_N(s)M_1 + b_N(s)N_1, \\ M_2 &= a_M(s)N_1 + b_M(s)M_2, \end{aligned} \tag{S17}$$

*with coefficients*

$$\begin{aligned} a_N(s) &= \frac{s\nu_M}{(\delta+\nu_M)(\delta+\nu_N) + s(2\delta+\nu_N+\nu_M)}, \\ b_N(s) &= \frac{(\delta+s+\nu_M)\nu_N}{(\delta+\nu_M)(\delta+\nu_N) + s(2\delta+\nu_N+\nu_M)}, \\ c_N(s) &= s+\delta+\nu_N\frac{\delta(\delta+\nu_M)+s(2\delta+\nu_M)}{(\delta+\nu_M)(\delta+\nu_N) + s(2\delta+\nu_N+\nu_M)}, \\ d(s) &= s\left(1+\frac{\nu_M\nu_N}{(\delta+\nu_M)(\delta+\nu_N) + s(2\delta+\nu_N+\nu_M)}\right), \\ A(s) &= \frac{(\nu_M-\nu_N)\delta(\delta+2s)}{s[(\nu_N\nu_M+(\delta+\nu_M)(\delta+\nu_N)+s(2\delta+\nu_N+\nu_M)]} \geq 0, \\ B(s) &= \frac{-A+\sqrt{A^2+4}}{2} \leq 1, \end{aligned} \tag{S18}$$

*and other follow from* $N \leftrightarrow M$ *symmetry. Importantly,* $M_1 = B(s)N_1$.

In the main text, we claimed that the diagonal of Fig. 3b corresponds to the population of uniform motility $\nu_N = \nu_M \equiv \nu$. A dynamical system for $(N+M)_{1,2}$ behaves as the basic source-sink model of Theorem 1, leading to a non-trivial fixed point

$$\begin{aligned} N_1 = M_1 &= \frac{K}{2}\left(1 - \frac{\delta(\delta+2\nu)}{r(\delta+\nu)}\right), \\ N_2 = M_2 &= \frac{\nu}{\nu+\delta}(N_1+N_2), \end{aligned}$$

which is unaffected by switching rate $s$. In the main text, we also showed that phenotypic switching can modulate the adaptation regime. This effect is controlled by the dimensionless number $A(s)$. As $A(s)$ contains only the $\delta, s$ rates (besides motilities), the low switching ($A \gg 1$, $s \ll \delta$) and high switching ($A \ll 1$, $s \gg \delta$) regimes can be distinguished and described.

**Corollary 2.** *At low switching (*$A \gg 1$, $s \ll \delta$*), the non-trivial fixed point of the source-sink model (*$L = 2, R = 1$*) is:*

$$\begin{aligned} N_1 w &= K\left(1 - \frac{\delta(\delta+2\nu_N)}{r(\delta+\nu_N)}\right) - \frac{s}{\delta}\frac{\nu_N\nu_M+(\delta+\nu_N)(\delta+\nu_M)}{\delta(\nu_M-\nu_N)}K\left(1 - \frac{\delta(\delta+2\nu_N)}{r(\delta+\nu_N)}\right) + \dots, \\ M_1 &= \frac{s}{\delta}K\left(1 - \frac{\delta(\delta+2\nu_N)}{r(\delta+\nu_N)}\right)\frac{\nu_N\nu_M+(\delta+\nu_N)(\delta+\nu_M)}{\delta(\nu_M-\nu_N)} + \dots, \\ N_2 &= \frac{\nu_N}{\delta+\nu_N}K\left(1 - \frac{\delta(\delta+2\nu_N)}{r(\delta+\nu_N)}\right)\left(1 - \frac{s}{\delta}\frac{(\delta+\nu_N)(\nu_N\nu_M+(\delta+\nu_N)(\delta+\nu_M))+\delta^2(\nu_M-\nu_N)}{\delta(\nu_M-\nu_N)(\delta+\nu_N)} + \dots\right), \\ M_2 &= \frac{s}{\delta}\frac{\nu_M}{\delta+\nu_M}K\left(1 - \frac{\delta(\delta+2\nu_N)}{r(\delta+\nu_N)}\right)\left(\frac{\nu_N\nu_M+(\delta+\nu_N)(\delta+\nu_M)}{\delta(\nu_M-\nu_N)} + \frac{\delta}{\delta+\nu_N}\right) + \dots, \end{aligned} \tag{S19}$$

*with the dimensionless coefficients*

$$
\begin{aligned}
A(s) &= \frac{\delta}{s} \frac{\delta(\nu_M - \nu_N)}{\nu_N \nu_M + (\delta + \nu_N)(\delta + \nu_M)} + \dots, \\
B(s) &= \frac{s}{\delta} \frac{\nu_N \nu_M + (\delta + \nu_N)(\delta + \nu_M)}{\delta(\nu_M - \nu_N)} + \dots .
\end{aligned}
\tag{S20}
$$

*In this case, the slower phenotype dominates the faster phenotype ($N_{1,2} \gg M_{1,2}$) and the system behaves as if only the slower phenotype was present ($(N+M)_{1,2}$ matches Theorem 1 with $\nu = \nu_N$).*

*At high switching ($A \ll 1$, $s \gg \delta$), the non-trivial fixed point of the source-sink model ($L = 2, R = 1$) is:*

$$
\begin{aligned}
N_1 &= \frac{K}{2}\left(1 - \frac{2\delta(\delta + \nu_M + \nu_N)}{r(2\delta + \nu_N + \nu_M)}\right) + \dots, \\
M_1 &= \frac{K}{2}\left(1 - \frac{2\delta(\delta + \nu_M + \nu_N)}{r(2\delta + \nu_N + \nu_M)}\right) + \dots, \\
N_2 &= \frac{\nu_N + \nu_M}{2\delta + \nu_N + \nu_M} \frac{K}{2}\left(1 - \frac{2\delta(\delta + \nu_M + \nu_N)}{r(2\delta + \nu_N + \nu_M)}\right) + \dots, \\
M_2 &= \frac{\nu_N + \nu_M}{2\delta + \nu_N + \nu_M} \frac{K}{2}\left(1 - \frac{2\delta(\delta + \nu_M + \nu_N)}{r(2\delta + \nu_N + \nu_M)}\right) + \dots,
\end{aligned}
\tag{S21}
$$

*with dimensionless coefficients*

$$
\begin{aligned}
A(s) &= \frac{\delta}{s} \frac{2(\nu_M - \nu_N)}{2\delta + \nu_N + \nu_M} + \dots, \\
B(s) &= 1 - \frac{\delta}{s} \frac{\nu_M - \nu_N}{2\delta + \nu_N + \nu_M} + \dots .
\end{aligned}
\tag{S22}
$$

*In this case, both phenotypes are similarly important ($N_{1,2} \approx M_{1,2}$) and the system behaves as if only an average phenotype was present ($(N+M)_{1,2}$ matches Theorem 1 with $\nu = (\nu_M + \nu_N)/2$).*

This result proves the following claim we made in the main text that at low switching ($A \ll 1$, $s \gg \delta$), the population adapts as if only the slower phenotype was present, and that at high switching ($A \ll 1$, $s \gg \delta$), the population adapts as if it had a uniform motility that matches the average motility.

In the main text, we also discussed the deadly motility regime. In contrast to systems with uniform motility, the deadly motility regime now occupies the $(\nu_N, \nu_M, s)$ space; and instead of critical motility, there is a critical surface in the $(\nu_N, \nu_M, s)$ space.

**Corollary 3.** *The deadly motility regime for the source-sink model ($L = 2, R = 1$) is described as a subspace of $(\nu_N, \nu_M, s)$ space such that:*

$$
D(s) > r - \delta, \tag{S23}
$$

*with*

$$
D(s) = s + \nu_N(1 - b_N(s)) - d(s)B(s). \tag{S24}
$$

*$D(s)$ has the following properties:*

- *$D(s)$ is a strictly increasing smooth function of $s \in \mathbb{R}^+$*
- $D(0) = \frac{\delta \nu_N}{\delta + \nu_N}$
- $D(\infty) = \frac{2\delta}{2\delta + \nu_N + \nu_M}$ *as $s \to \infty$*

*The deadly motility regime exist whenever the environment is sink-like $\overline{f} = (r - 2\delta)/2 < 0$, and*

- *the slower phenotype is in the deadly motility regime $\min(\nu_N, \nu_M) > \nu_c$, and switching $s$ is arbitrary, or*
- *the average phenotype is in the deadly motility regime $(\nu_N + \nu_M)/2 > \nu_c$, and switching $s$ is above some critical value $s > s_c(\nu_N, \nu_M)$.*

*In particular, the critical surface asymptotes with* $\min(\nu_N, \nu_M) = \nu_c$ *at low switching* ($s \ll \delta$) *and with* $(\nu_N + \nu_M)/2 = \nu_c$ *at high switching* ($s \gg \delta$).

For completeness, we state the results for the staircase model, which can be used to support the same claims as those made above for the simpler source-sink model. The asymptotic form of the wild-type profile can be derived.

**Theorem 7.** *In the low motility combination* $\nu_N, \nu_M \ll \delta$*, the non-trivial fixed point of the staircase model is given by:*

$$\begin{aligned} M_x &= \begin{cases} \frac{K}{2}\left(1-\frac{\delta}{r}\right)+\dots & \text{if } x=1,\dots,R-1 \\ \frac{K}{2}\left(1-\frac{\delta+(\nu_N+\nu_M)/2}{r}\right)-\frac{K}{8s}\left(1-\frac{\delta}{r}\right)(\nu_M-\nu_N)+\dots & \text{if } x=R \\ \frac{K}{4}\left(1-\frac{\delta}{r}\right)\left(\frac{\nu_N+\nu_M}{\delta}+\frac{\nu_M-\nu_N}{\delta+2s}\right)++\dots & \text{if } x=R+1 \\ 0+\dots & \text{if } x=R+2,\dots,L \end{cases} \\ N_x &= \begin{cases} \frac{K}{2}\left(1-\frac{\delta}{r}\right)+\dots & \text{if } x=1,\dots,R-1 \\ \frac{K}{2}\left(1-\frac{\delta+(\nu_N+\nu_M)/2}{r}\right)-\frac{K}{8s}\left(1-\frac{\delta}{r}\right)(\nu_N-\nu_M)+\dots & \text{if } x=R \\ \frac{K}{4}\left(1-\frac{\delta}{r}\right)\left(\frac{\nu_N+\nu_M}{\delta}+\frac{\nu_N-\nu_M}{\delta+2s}\right)++\dots & \text{if } x=R+1 \\ 0+\dots & \text{if } x=R+2,\dots,L \end{cases} \end{aligned} \tag{S25}$$

*In the high motility combination* $\nu_N, \nu_M \gg \delta$*, the non-trivial fixed point of the staircase model is given by:*

$$N_x = M_x = \frac{K}{2}\left(1-\frac{L\delta}{Rr}\right)+\dots. \tag{S26}$$

*In the mixed motility combination* $\nu_N \ll \delta \ll \nu_M$*, the non-trivial fixed point of the staircase model is given by*

$$\begin{aligned} M_x &= K\left(1-\frac{\delta+s(1-B(s))}{r}\right)\frac{B(s)}{1+B(s)}+\dots \\ N_x &= \begin{cases} K\left(1-\frac{\delta+s(1-B(s))}{r}\right)\frac{1}{1+B(s)}+\dots & \text{if } x\le R \\ K\left(1-\frac{\delta+s(1-B(s))}{r}\right)\frac{sB(s)}{(1+B(s))(s+\delta)}+\dots & \text{if } x>R \end{cases} \end{aligned} \tag{S27}$$

*with*

$$\begin{aligned} A(s) &= \left(\frac{L}{R}-1\right)\frac{\delta^2+2\delta s}{s^2+\delta s}>0, \\ B(s) &= \frac{-A+\sqrt{A^2+4}}{2}<1. \end{aligned} \tag{S28}$$

*Importantly, to lowest order* $N_x/M_x = B(s)$ *in the region above the staircase* $x \ge R$.

A non-perturbative result characterising the behaviour of the staircase model at low and high switching can also be derived analytically.

**Theorem 8.** *At low switching* $s \ll \delta$*, the overall wild-type profile behaves as if it has a single motility of* $\nu = \min(\nu_N, \nu_M)$*. At high switching* $s \gg \delta$*, the overall wild-type profile behaves as if it has a single motility of* $\nu = (\nu_N + \nu_M)/2$.

*In particular, this result affects the deadly motility regime in the* $(\nu_N, \nu_M, s)$ *space. Let* $\overline{f} = (rR - \delta L)/L$ *be the average wild-type fitness. The wild-type profile is non-trivial iff:*

- *the environment is source-like* $\overline{f} > 0$*, motility rates* $\nu_N, \nu_M$ *are arbitrary, and switching rate* $s$ *is arbitrary,*
- *the environment is sink-like* $\overline{f} < 0$ *and*
  - *switching is low* $s \ll \delta$ *and the slower phenotype is in the deadly motility regime* $\min(\nu_N, \nu_M) > \nu_c$*,*
  - *switching is high* $s \gg \delta$ *and the average-motility phenotype is in the deadly motility regime* $(\nu_N+\nu_M)/2 > \nu_c$.

**Supplementary Note 6. Density-dependent motility.**

*1. Model with implicit phenotypic structure.*

In the main text, we studied the wild-type profiles of populations with density-dependent motility, where motility phenotypes were modelled implicitly and the evolving population at a given spatial position $x$ was approximated by a single motility phenotype. We briefly outline important features of these wild-type profiles. The wild-type profile can be described with the mean-field equations:

$$\begin{aligned}
\dot{N}_1 &= r\left(1-\frac{N_1}{K}\right)N_1 && + \nu(N_2)N_2 && -(\nu(N_1)+\delta)N_1, && \\
\dot{N}_x &= r\left(1-\frac{N_x}{K}\right)N_x && +\nu(N_{x-1})N_{x-1}+\nu(N_{x+1})N_{x+1} && -(2\nu(N_x)+\delta)N_x,\ x && = 2,\dots,R \\
\dot{N}_x &= && \nu(N_{x-1})N_{x-1}+\nu(N_{x+1})N_{x+1} && -(2\nu(N_x)+\delta)N_x,\ x && = R+1,\dots,L-1 \\
\dot{N}_L &= && \nu(N_{L-1})N_{L-1} && -(\nu(N_x)+\delta)N_L., &&
\end{aligned} \tag{S29}$$

where

$$\nu(N) = \begin{cases} \nu_L & \text{if } N < S, \\ \nu_H & \text{if } N \geq S. \end{cases} \tag{S30}$$

Since the forcing of this dynamical system is discontinuous whenever $N_x = S$, the fixed-point analysis is more complicated and we only mention two important features of this system.

First, multiple stable fixed points can exist. For example, consider a situation when a low-density motility phenotype has motility above the critical motility $\nu_L > \nu_c$ and the high-density motility phenotype is slower $\nu_H < \nu_L$. If the system is initially started from a low density, the low-density motility phenotype cannot survive and a deadly motility regime occurs as in Fig. 4c (attraction to the trivial fixed-point). However, if the system is initially started from a high density, the high-density motility phenotype can survive and no deadly motility regime would have occured in Fig. 4c (attraction to a non-trivial fixed-point).

Second, the surfaces of discontinuous forcing ($N_x = S$ for some $x$) might attract the trajectories. This result often happens in the slow-to-fast switching case, see Fig. 4b at low threshold $S$. In this case, the bulk of the high-density population is driven very close to density $N_x = S$.

*2. Model with explicit phenotypic structure.*

Density-dependent motility can also be modelled explicitly at each position $x$, but explicit density-dependent motility leads to qualitatively similar conclusions. Here, we explain such modelling and why one obtains similar results from models with implicit and explicit phenotypic structure. To introduce the explicit model, we consider the same setup as in the model for stochastic motility switching (Fig. 3a) where two phenotypes of different motility $\nu_{1,2}$ are introduced and switched at rate $s$. To introduce density-dependent motility, we modify the switching rates $s$ between the motility phenotypes $P$ (Supplementary Fig. 8a) In particular, at a given spatial position $x$ with $N_x$ cells, phenotype 1 switches into phenotype 2 at a rate

$$s_{1\to 2} = \begin{cases} s\beta/(1+\beta) & \text{if } N_x < S, \\ s/(1+\beta) & \text{if } N_x \geq S, \end{cases} \tag{S31}$$

and phenotype 2 switches into phenotype 1 at a rate

$$s_{2\to 1} = \begin{cases} s/(1+\beta) & \text{if } N_x < S, \\ s\beta/(1+\beta) & \text{if } N_x \geq S. \end{cases} \tag{S32}$$

The total switching rate $s_{1\to 2} + s_{2\to 1} = 2s$ is kept constant and the phenotypes switch $\beta$-times more likely in the direction $1 \to 2$ than $2 \to 1$ at low density ($N_x < S$), while they switch $\beta$-times more likely in the direction $2 \to 1$ than $1 \to 2$ at high density ($N_x > S$). To identify phenotype 1 with the low-density phenotype ($\nu_1 = \nu_L$) and phenotype 2 with the high-density phenotype ($\nu_2 = \nu_H$), we restrict to $\beta \leq 1$.

To start the analysis of this model, we notice a few limiting cases. If the total switching is low ($s \ll \delta$), phenotypic switching is rare and the model is expected to behave similarly to the model for stochastic switching with low rate $s$, reasoned from the same arguments as in Supplementary Note 5. Therefore, to explore switching, we restrict to situations when the switching rate is high ($s \gg \delta$). Moreover, if no phenotype is preferred ($\beta = 1$), the model is equivalent to the model of stochastic switching. Therefore, to explore density-dependence, we restrict to $\beta \ll 1$. In summary, we expect density-dependent switching to be important only if $s \gg \delta$ and $\beta \ll 1$. The implicit model of density-dependent switching corresponds to $s \to \infty$ and $\beta \to 0$. Therefore, the implicit model is expected to exhibit the same dynamics as the explicit model for density-dependent switching.

Indeed, the evolutionary dynamics is governed by the same effective motility in both models. The effective motility corresponds to the low-density motility $\nu_L$ at high switching threshold $S \gg K(1-\delta/r)$ and to the high-density motility $\nu_H$ at low switching threshold $S \ll K(1-\delta/r)$ (Supplementary Fig. 8d,e). Moreover, the wild-type profiles are very similar and differ only in predicted phenotypic proportions at individual spatial positions (Supplementary Fig. 8f). While explicit density-dependent motility is more realistic, we note, however, that the implicit model is faster to simulate, and, therefore, it is presented in the main text.

### Supplementary Note 7. Linking theory and experiments.

To explain the link between our theory and experiments, we introduce the concept of visiting number, which is a non-dimensional number that captures the drug variability experienced by an average cell during its lifetime. Specifically, the visiting number $V$ is defined as the number of regions that differ in drug concentrations (on MIC scale, MIC = minimal inhibitory concentration) and that are visited by an average cell during its life-time. In the staircase model, the probability that a cell moves between $k$ such regions in its lifetime is

$$p_k = \left(\frac{2\nu}{2\nu + r_e + \delta + \mu_f + \mu_b}\right)^k \left(\frac{r_e + \delta + \mu_f + \mu_b}{2\nu + r_e + \delta + \mu_f + \mu_b}\right),$$

where $r_e = r(1 - N_x/K)$ is the effective division rate. This is a geometric probability distribution with a known mean that corresponds to the visiting number $V$. On average, a cells visits

$$V = \frac{2\nu}{\delta + r_e + \mu_f + \mu_b} \approx \frac{\nu}{\delta}$$

compartments that differ in the level of MIC (where we used the fact that the effective division rate $r_e = r(1-N_x/K)$ approximately balances the death rate $\delta$ at equilibrium). Consequently, it can be noticed that the low motility regime corresponds to $\nu < \delta$ (i.e., $V < 1$) and the high motility regime to $\nu > \delta$ (i.e., $V > 1$). While $\nu$ and $\delta$ are difficult to measure in practice, the visiting number $V$ can be estimated as,

$$V = \frac{vt}{l},$$

where $v$ is the characteristic cell speed, $t$ is the doubling time in the absence of antibiotics and $l$ is the length-scale over which drug concentrations vary on MIC scales. This expression follows from the fact that an average cell explores the length scale of $vt$ during its life span, while it visits $vt/l$ regions of length scale $l$. To link our models with experimental works explicitly, we estimate the visiting numbers in experiments[S21,S22] and predict their motility regime (Supplementary Table 1). To clarify, the length scale $l$ is taken directly from Baym *et al.*[S21] and Zhang *et al.*,[S22] while the speed $v$ and doubling time $t$ was not measured in these works. However, Baym *et al.*[S21] and Zhang *et al.*[S22] used *E. coli* K-12, which is known to swim at 10$\mu$m/s[S23,S24] and its doubling time is estimated at 40min.[S25] Next, we look for qualitative features of the reported evolutionary dynamics and compared them to our model, namely Fig. 1d. Notably, we find that there is an agreement between the value of the visiting number ($V \gtrless 1$) and the qualitative features of the associated motility regime predicted by our model (Fig. 1d, Supplementary Table 1).

### Supplementary Note 8. Survival Probability.

This Supplementary Note explains how to compute the survival probability $q$ of the first mutant in the overlap region, i.e., the probability that this mutant divides before its death. To start, we define the probability $q_x$ that a mutant divides before its death, given that the mutant starts in spatial compartment $x$. Mutants in compartment $x$ can change their genotype (rate $\mu_f + \mu_b$), die (rate $\delta_x$), move left (rates $\nu_{L,x-1}$), move right ($\nu_{R,x}$) or divide (rate $r_x$).

**Supplementary Table** 1 | Experimental examples of different adaptation regimes explored in our work.

| experiment | v | t | l | V | adaptation regime | qualitative evidence for the regime |
|---|---|---|---|---|---|---|
| MEGA-plate[S21] | 10$\mu$m/s[S23,S24] | 40min[S25,S26] | 13cm | 0.1846 | low motility regime | adaptation dynamics located at the front, coexistence of resistance strains |
| microenvironments[S22] | 10$\mu$m/s[S23,S24] | 40min[S25,S26] | 200$\mu$m | 120 | high motility regime | resistant mutants invade the entire environment and replace the wild-type |

Therefore, the total rate at which the mutant experiences one of the processes is $\rho_x = \mu_f + \mu_b + \delta_x + \nu_{L,x-1} + \nu_{R,x} + r_x$. To find $q_x$, we condition on the first process to obtain that

$$q_x = \mathbb{1}_{x>1} \frac{\nu_{L,x-1}}{\rho_x} q_{x-1} + \mathbb{1}_{x<L} \frac{\nu_{R,x}}{\rho_x} q_{x+1} + \frac{r_x}{\rho_x}. \tag{S33}$$

This is a system of linear algebraic equations for $q_x$ that can be solved numerically, e.g., by Gaussian elimination method. The survival probability of the mutant that started in the overlap region is $q = q_{R+1}$.

This survival probability must be accounted for in the computation of the evolutionary time $T^R_{\text{evo}}$ whenever $q \ll 1$. If the first mutant in the overlap region dies before dividing, a new mutant must arrive into the overlap region. This process takes additional evolutionary time $T^R_{\text{evo}}$. Moreover, the probability that $k$ mutants arrive into the overlap region before one of them successfully divides is $(1-q)^{k-1}q$. Finally, the expected time, till a mutant that divides before death is produced in the overlap region, is

$$\sum_{k=1}^{\infty} (1-q)^{k-1} q \mathbb{E}(k T^R_{\text{evo}}) = \mathbb{E} T^R_{\text{evo}}/q. \tag{S34}$$

## Supplementary Note 9. Proofs.

**Theorem 1.** *System with equation* (4) *admits exactly two fixed points*

- *trivial fixed point:* $N_2 = N_1 = 0$,
- *non-trivial fixed point:*

$$\begin{aligned} N_1 &= K\left(1 - \frac{\delta(\delta+2\nu)}{r(\delta+\nu)}\right), \\ N_2 &= \frac{\nu}{\nu+\delta} N_1. \end{aligned} \tag{S1}$$

*Exactly one of these fixed points is stable and corresponds to a stable wild-type profile, with non-negative cell numbers* $N_{1,2} \geq 0$. *Let* $\overline{f} = (r - 2\delta)/2$ *be the average wild-type fitness. The stable wild-type profile corresponds to the non-trivial fixed point iff*

- *the environment is source-like* $\overline{f} > 0$ *and cell motility* $\nu$ *is arbitrary, or*
- *the environment is sink-like* $\overline{f} < 0$ *and cell motility is constrained to* $\nu \in [0, \nu_c]$, *where the critical motility is*

$$\nu_c = \frac{\delta(r-\delta)}{2\delta - r} > 0. \tag{S2}$$

*Proof.* The two fixed points can be found by setting $\dot{N}_1 = \dot{N}_2 = 0$. The stability of these fixed points can be checked by considering the signs of trace and determinant of the Jacobian matrix:

$$J = \begin{pmatrix} r - 2rN_1/K - \delta - \nu & \nu \\ \nu & -\delta - \nu \end{pmatrix}. \tag{S35}$$

For example, the trivial fixed point has tr $J = r - 2\delta - 2\nu$ and det $J = -\delta(r-\delta) + \nu(2\delta - r)$. If $\overline{f} > 0$, or if $\overline{f} < 0$ and $\nu < \nu_c$, then det $J < 0$ and the trivial fixed point is a saddle node. Otherwise, det $J > 0$, tr $J < 0$ and the trivial fixed point is stable. Similar analysis applies to the non-trivial fixed point. □

**Theorem 2.** *Let $N_x$ be any fixed point of the dynamical system with equation* (S6) *that lies in the strictly positive quadrant $N_x > 0$. Note that $N_x$ coincides with the non-trivial fixed point $N_x$ if it is stable. This wild-type profile $N_x$ of resistance $R$ satisfies:*

1. *$N_x$ is a decreasing function of $x$.*
2. *In any compartment above the staircase, the effective wild-type birth rate $r(1 - N_x/K)$ is bigger than the death rate $\delta$.*
3. *$N_x$ is concave above the staircase, i.e., on $[1, R+1]$.*
4. *$N_x$ is convex below the staircase, i.e., on $[R, L]$.*
5. *The curvature of the wild-type population profile at position $x$ is proportional to wild-type fitness $r(1 - N_x/K)N_x\mathbb{1}_{x\le R} - \delta N_x$ at this position and inversely proportional to motility.*
6. *The mutant fitness $r(1 - N_x/K)N_x\mathbb{1}_{x\le R+1} - \delta N_x$ is a positive, convex and increasing function on $[1, R+1]$, and a constant $-\delta$ function on $[R+2, L]$. In particular, mutant fitness is maximised in the overlap region and matches the wild-type fitness characterised above in compartments $x \le R$.*
7. *In any compartment $N_x < K(1 - \delta/r)$.*
8. *$r > \delta$ is a necessary condition for the existence of this non-trivial fixed point in the strictly positive quadrant.*

*Proof. Part 1 and 2:* Rewrite the fixed point equation for the dynamical system with equation (S6) as

$$\begin{aligned} N_1 - N_2 &= \frac{N_1}{\nu}\left[r\left(1 - \frac{N_1}{K}\right) - \delta\right] \\ N_x - N_{x+1} &= \frac{N_x}{\nu}\left[r\left(1 - \frac{N_x}{K}\right) - \delta\right] + N_{x-1} - N_x, \qquad x = 2, \dots, R \\ N_x - N_{x+1} &= -\frac{\delta N_x}{\nu} + N_{x-1} - N_x, \qquad x = R+1, \dots, L-1 \\ N_{L-1} - N_L &= \frac{\delta N_L}{\nu}. \end{aligned}$$

Firstly, assume that $N_1 \le N_2$. The first equation implies that $r(1-N_1/K)-\delta \le 0$. As $N_1 \le N_2$, also $r(1-N_2/K)-\delta \le r(1-N_1/K)-\delta \le 0$. Then, the second equation implies that $N_2 \le N_3$. Inductively, the first $L-1$ equations imply that

$$N_1 \le \cdots \le N_x \le N_{x+1} \le \cdots \le N_L.$$

However, the last equations then implies that $N_L \le 0$, contradicting our restriction to $N_x > 0$. Therefore, $N_1 > N_2$. The first equation implies that $r(1 - N_1/K) - \delta > 0$. As $N_1 > N_2$, $r(1 - N_2/K) - \delta > r(1 - N_1/K) - \delta > 0$. The second equation implies that $N_2 - N_3 > 0$. Inductively, the first $L-1$ equations imply the conclusion of part 1 that

$$N_1 > \cdots > N_x > N_{x+1} > \cdots > N_L.$$

Notice that along the way, part 2 has also been proven.

*Part 3:* Notice that above the staircase,

$$\frac{N_{x+1} + N_{x-1}}{2} - N_x = -\frac{N_x}{2\nu}\left[r\left(1 - \frac{N_x}{K}\right) - \delta\right] < 0.$$

*Part 4:* Notice that below the staircase,

$$\frac{N_{x+1} + N_{x-1}}{2} - N_x = \frac{N_x\delta}{2\nu} > 0.$$

*Part 5:* Write the fixed point equation in the form:

$$
\begin{aligned}
-\nu(N_2 - N_1) &= \left[r\left(1-\frac{N_1}{K}\right)-\delta\right]N_1 \\
\nu\left[N_{x+1}+N_{x-1}-2N_x\right] &= \left[r\left(1-\frac{N_x}{K}\right)-\delta\right]N_x, \qquad x=2,\dots,R \\
\nu\left[N_{x+1}+N_{x-1}-2N_x\right] &= -\delta N_x, \qquad\qquad\qquad\qquad x=R+1,\dots,L \\
-\nu(N_{L-1}-N_L) &= -\delta N_L.
\end{aligned}
$$

*Part 6:* The mutant fitness is given by the difference between the effective birth-rate (resulting from competition with wild-type) and death rate. Therefore, on $[1, R+1]$, this mutant fitness is

$$
r\left(1-\frac{N_x}{K}\right)-\delta.
$$

Therefore, it is increasing (by part 1), positive (by part 2) and convex (by part 3). On $[R+2, L]$, mutants cannot divide and their fitness is $-\delta$.

*Part 7:* Follows directly from part 2.

*Part 8:* If $r \leq \delta$, part 6 implies that $N_x \leq 0$. □

**Theorem 3.** *In the low motility regime $\nu \ll \delta$, the non-trivial fixed point is given by:*

$$
N_x = \begin{cases} K\left(1-\frac{\delta}{r}\right) + O\left(\left(\frac{\nu}{\delta}\right)^2\right) & \text{if } x = 1,\dots,R-1 \\ K\left(1-\frac{\delta+\nu}{r}\right) + O\left(\left(\frac{\nu}{\delta}\right)^2\right) & \text{if } x = R \\ K\frac{\nu}{\delta}\left(1-\frac{\delta}{r}\right) + O\left(\left(\frac{\nu}{\delta}\right)^2\right) & \text{if } x = R+1 \\ 0 + O\left(\left(\frac{\nu}{\delta}\right)^2\right) & \text{if } x = R+2,\dots,L \end{cases} \tag{S7}
$$

*In the high motility regime $\nu \gg \delta$, the non-trivial fixed point is given by:*

$$
N_x = K\left(1-\frac{\delta L}{rR}\right) + b_x\frac{\delta}{\nu} + O\left(\left(\frac{\delta}{\nu}\right)^2\right), \tag{S8}
$$

*where the coefficients $b_x$ satisfy*

$$
\begin{aligned}
b_1 &= \frac{\delta K}{6rR}\left(3L^3 - 3L^2R + 3LR^2 + (R-L)(R^2-1)\left(\frac{2L}{R}-\frac{r}{\delta}\right)\right), \\
b_x &= b_1 + K\left(1-\frac{\delta L}{rR}\right)\frac{x(x-1)}{2}\left(1-\frac{L}{R}\right), \text{ if } x = 2,\dots,R+1, \\
b_x &= b_1 + K\left(1-\frac{\delta L}{rR}\right)\left(\frac{x(x-1)+L(1+R-2x)}{2}\right), \text{ if } x = R,\dots,L.
\end{aligned} \tag{S9}
$$

*Proof.* For the low motility regime, assume that $N_x = a_x + \frac{\nu}{\delta}b_x + \dots$ Then, at $O(1)$ of equation (S6), we recover the equilibrium of local birth/death dynamics:

$$
\begin{aligned}
r\left(1-\frac{a_x}{K}\right)a_x &= \delta a_x, \qquad x = 1,2,\dots,R \\
0 &= \delta a_x, \qquad x = R+1,\dots,L
\end{aligned}
$$

implying that $a_x = K(1-\delta/r)$ when $x \leq R$, and $a_x = 0$ otherwise. At $O(\nu/\delta)$, we recover the first order corrections to balance this local dynamics with curvature of the population curve:

$$
\begin{aligned}
\frac{r}{\delta}\left(1-\frac{2a_1}{K}\right)b_1 + a_2 \qquad\qquad &= b_1 + a_1 \\
\frac{r}{\delta}\left(1-\frac{2a_x}{K}\right)b_x + a_{x+1} \quad + a_{x-1} &= b_x + 2a_x, \qquad x = 2,\dots,R \\
+\, a_{x+1} \quad + a_{x-1} &= b_x + 2a_x, \qquad x = R+1,\dots,L-1 \\
+ a_{L-1} &= b_L + a_L.
\end{aligned}
$$

Using the previous formulae for $a_x$ this can be solved for $b_x$. The result follows.

For the high motility regime, assume that $N_x = a_x + \frac{\delta}{\nu} b_x + \left(\frac{\delta}{\nu}\right)^2 c_x + \dots$ Then, at $O(\left(\frac{\delta}{\nu}\right)^{-1})$, we only get the spatial diffusion with neglected local birth-death processes:

$$\begin{aligned} a_1 &= a_2 \\ a_{x+1} + a_{x-1} &= a_x, \quad x = 2, \dots, L-1 \\ a_{L-1} &= a_L. \end{aligned}$$

This result implies that $a_x = a$ for some constant $a$. Also notice that this system is under-determined and constant $a$ must be fixed from the equations at the next order. At $O(1)$, the first order correction to curvature due to local birth/death processes is:

$$\begin{aligned} \frac{r}{\delta}\left(1 - \frac{a}{K}\right) a + b_2 \qquad\qquad &= b_1 + a \\ \frac{r}{\delta}\left(1 - \frac{a}{K}\right) a + b_{x+1} \quad + b_{x-1} &= 2b_x + a, \quad x = 2, \dots, R \\ + b_{x+1} \quad + b_{x-1} &= 2b_x + a, \quad x = R+1, \dots, L-1 \\ + b_{L-1} &= b_L + a. \end{aligned}$$

Summing the equations yields

$$R\frac{r}{\delta}\left(1 - \frac{a}{K}\right) a = La,$$

and thus

$$a = K\left(1 - \frac{\delta L}{rR}\right).$$

With this choice of $a$, the system of equations for $b_x$ takes a form of under-determined system of difference equations. By solving this system, $b_x$'s can can be expressed in terms of $b_1$ as in equation (S9). In order to fix $b_1$, the equations for $c_x$ at order $O(\frac{\delta}{\nu})$ need to be found:

$$\begin{aligned} \frac{r}{\delta}\left(1 - \frac{2a}{K}\right) b_1 + c_2 \qquad\qquad &= b_1 + c_1 \\ \frac{r}{\delta}\left(1 - \frac{2a}{K}\right) b_x + c_{x+1} \quad + c_{x-1} &= b_x + 2c_x, \quad x = 2, \dots, R \\ + c_{x+1} \quad + c_{x-1} &= b_x + 2c_x, \quad x = R+1, \dots, L-1 \\ + c_{L-1} &= b_L + c_L. \end{aligned}$$

Summation cancels the $c_x$ coefficients and gives a single equation for $b_x$:

$$\frac{r}{\delta}\left(1 - \frac{2a}{K}\right)(b_1 + \dots + b_R) = b_1 + \dots + b_L.$$

Using the relationships for $b_x$:

$$b_1 + \dots + b_R = Rb_1 + \frac{a}{6}(R^2 - 1)(R - L),$$

and

$$b_1 + \dots + b_L = Lb_1 + \frac{a}{6}L(-3L^2 - R^2 + 3LR),$$

the coefficient $b_1$ is obtained as in equation (S9). □

**Corollary 1.** *Let* $\overline{f} = (rR - \delta L)/L$ *be the average wild-type fitness. Then, the stable wild-type profile is non-vanishing iff*

- *the environment is source-like* $\overline{f} > 0$ *and motility* $\nu$ *is arbitrary, or*

- *the environment is sink-like* $\overline{f} < 0$ *and motility is constrained to* $\nu \in [0, \nu_c]$*, where to first order*

$$\nu_c = \frac{\delta b_L}{K\left(\frac{\delta L}{rR} - 1\right)}. \tag{S10}$$

*Proof.* Notice that a stable wild-type profile $N_x$ is expected to be decreasing in $x$ by Theorem 2. Hence, a non-trivial fixed point stays within the positive quadrant and remains stable iff $N_L > 0$. To the lowest order, this condition is equivalent to $\overline{f} > 0$. If $\overline{f} > 0$, the wild-type profile exists for any $\nu$. If $\overline{f} < 0$, the first order approximation dictates that $N_L > 0$ iff $\nu < \nu_c$ with $\nu_c$ given by equation (S10). At $\nu = \nu_c$, the non-trivial wild-type fixed point collides with the trivial wild-type fixed point and exchanges stability through a transcritical bifurcation. □

**Theorem 4.** *Fix a resistance state* $R$ *and define* $a_{evo} = 1/\mathbb{E}T^R_{evo}$*. Then,*

$$a_R < a_{evo} < \mu_f \sum_x N_x,$$

*In particular, when critical motility* $\nu_c$ *exists, the adaptation rate* $a_R$ *decreases to* 0 *faster than* $\mu_f \sum_x N_x$ *as* $\nu \to \nu_c^-$*. Moreover, no adaptation can occur if the wild-type population is extinct.*

*Proof.* To find the upper bound on $a_{\text{evo}}$, we need to find the lower bound on the waiting time $T^R_{\text{evo}}$ till the stable wild-type population produces a mutant in the overlap region (mutants migrating from other compartments count as well). This waiting time $T^R_{\text{evo}}$ is bounded below by the waiting time till a mutant is produced anywhere $T_a$, i.e., $T^R_{\text{evo}} > T_a$. In each spatial compartment $x$, only the most resistant wild-type cells can mutate. Call their number $N'_x$. Then, the waiting time $T_x$ till the compartment $x$ produces a mutant is $\sim \text{Exp}(\mu_f N'_x)$. The waiting times $T_x$ are independent and $T_a = \min_x T_x$. Therefore, $T_a \sim \text{Exp}(\mu_f \sum_x N'_x)$. Finally,

$$\mathbb{E}T^R_{\text{evo}} > \mathbb{E}T_a = \frac{1}{\mu_f \sum_x N'_x}.$$

Thus,

$$a_{\text{evo}} = \frac{1}{\mathbb{E}T^R_{\text{evo}}} < \mu_f \sum_x N'_x \leq \mu_f \sum_x N_x = \mu_f N.$$

Result follows. □

**Theorem 6.** *The dynamical system with equation* (S16) *for the source-sink model* ($L = 2, R = 1$) *has two fixed points:*

- *the trivial fixed point:* $N_{1,2} = M_{1,2} = 0$*,*

- *the non-trivial fixed point:*

$$\begin{aligned}
N_1 &= \frac{K}{1 + B(s)} \left(1 - \frac{c_N(s) - d(s)B(s)}{r}\right), \\
M_1 &= \frac{K}{1 + B(s)^{-1}} \left(1 - \frac{c_M(s) - d(s)B(s)^{-1}}{r}\right), \\
N_2 &= a_N(s)M_1 + b_N(s)N_1, \\
M_2 &= a_M(s)N_1 + b_M(s)M_2,
\end{aligned} \tag{S17}$$

*with coefficients*

$$
\begin{aligned}
a_N(s) &= \frac{s\nu_M}{(\delta+\nu_M)(\delta+\nu_N)+s(2\delta+\nu_N+\nu_M)},\\
b_N(s) &= \frac{(\delta+s+\nu_M)\nu_N}{(\delta+\nu_M)(\delta+\nu_N)+s(2\delta+\nu_N+\nu_M)},\\
c_N(s) &= s+\delta+\nu_N\frac{\delta(\delta+\nu_M)+s(2\delta+\nu_M)}{(\delta+\nu_M)(\delta+\nu_N)+s(2\delta+\nu_N+\nu_M)},\\
d(s) &= s\left(1+\frac{\nu_M\nu_N}{(\delta+\nu_M)(\delta+\nu_N)+s(2\delta+\nu_N+\nu_M)}\right),\\
A(s) &= \frac{(\nu_M-\nu_N)\delta(\delta+2s)}{s[(\nu_N\nu_M+(\delta+\nu_M)(\delta+\nu_N)+s(2\delta+\nu_N+\nu_M)]}\geq 0,\\
B(s) &= \frac{-A+\sqrt{A^2+4}}{2}\leq 1,
\end{aligned}
\tag{S18}
$$

*and other follow from $N \leftrightarrow M$ symmetry. Importantly, $M_1 = B(s)N_1$.*

*Proof.* Notice that the system with equation (S16) is symmetric under $M \leftrightarrow N$. Now, express $M_2, N_2$ in terms of $N_1$, $M_1$ using the equations $\dot{N}_2 = 0$ and $\dot{M}_2 = 0$:

$$
\begin{aligned}
N_2 &= a_N(s)M_1 + b_N(s)N_1,\\
M_2 &= a_M(s)N_1 + b_M(s)M_1,
\end{aligned}
$$

with the coefficients

$$
\begin{aligned}
a_N(s) &= \frac{s\nu_M}{(\delta+\nu_M)(\delta+\nu_N)+s(2\delta+\nu_N+\nu_M)},\\
b_N(s) &= \frac{(\delta+s+\nu_M)\nu_N}{(\delta+\nu_M)(\delta+\nu_N)+s(2\delta+\nu_N+\nu_M)},
\end{aligned}
$$

and $a_M(s), b_M(s)$ follow by $N \leftrightarrow M$. This changes the the equations $\dot{N}_1 = 0$ and $\dot{M}_1 = 0$ into

$$
\begin{aligned}
0 &= r(1-(N_1+M_1)/K)N_1 - c_N(s)N_1 + d(s)M_1,\\
0 &= r(1-(N_1+M_1)/K)M_1 - c_M(s)M_1 + d(s)N_1,
\end{aligned}
$$

with coefficients

$$
\begin{aligned}
c_N(s) &= s+\delta+\nu_N(1-b_N(s)) = s+\delta+\nu_N\frac{\delta(\delta+\nu_M)+s(2\delta+\nu_M)}{(\delta+\nu_M)(\delta+\nu_N)+s(2\delta+\nu_N+\nu_M)},\\
d(s) &= s+\nu_N a_N(s) = s\left(1+\frac{\nu_M\nu_N}{(\delta+\nu_M)(\delta+\nu_N)+s(2\delta+\nu_N+\nu_M)}\right),
\end{aligned}
$$

and $c_M(s)$ follows by $N \leftrightarrow M$. Multiplying the first equation by $M_1$, the second equation by $N_2$ and subtracting, we obtain

$$
(c_M(s) - c_N(s))M_1N_1 = d(s)(N_1^2 - M_1^2).
$$

Therefore, either $N_1 = 0$ or $N_1 \neq 0$. In the first case, we obtain the trivial fixed point by following back through the equations. In the latter case,

$$
\frac{N_1}{M_1} - \frac{M_1}{N_1} = \frac{c_M(s)-c_N(s)}{d(s)} \equiv A(s),
$$

with

$$
A(s) = \frac{(\nu_M-\nu_N)\delta(\delta+2s)}{s[(\nu_N\nu_M+(\delta+\nu_M)(\delta+\nu_N)+s(2\delta+\nu_N+\nu_M)]} \geq 0
$$

Therefore,

$$
\frac{M_1}{N_1} = \frac{-A+\sqrt{A^2+4}}{2} \equiv B(s) \leq 1.
$$

Finally, this result can be used to derive:

$$N_1 = \frac{K}{1+B(s)}\left(1 - \frac{c_N(s) - d(s)B(s)}{r}\right).$$

We also note that the symmetry $N \leftrightarrow M$ is preserved as

$$M_1 = B(s)N_1 = \frac{K}{1+B(s)^{-1}}\left(1 - \frac{c_M(s) - d(s)B(s)^{-1}}{r}\right).$$

□

**Corollary 3.** *The deadly motility regime for the source-sink model ($L = 2, R = 1$) is described as a subspace of $(\nu_N, \nu_M, s)$ space such that:*

$$D(s) > r - \delta, \tag{S23}$$

*with*

$$D(s) = s + \nu_N(1 - b_N(s)) - d(s)B(s). \tag{S24}$$

*$D(s)$ has the following properties:*

- *$D(s)$ is a strictly increasing smooth function of $s \in \mathbb{R}^+$*
- *$D(0) = \frac{\delta\nu_N}{\delta+\nu_N}$*
- *$D(\infty) = \frac{2\delta}{2\delta+\nu_N+\nu_M}$ as $s \to \infty$*

*The deadly motility regime exist whenever the environment is sink-like $\overline{f} = (r - 2\delta)/2 < 0$, and*

- *the slower phenotype is in the deadly motility regime $\min(\nu_N, \nu_M) > \nu_c$, and switching $s$ is arbitrary, or*
- *the average phenotype is in the deadly motility regime $(\nu_N + \nu_M)/2 > \nu_c$, and switching $s$ is above some critical value $s > s_c(\nu_N, \nu_M)$.*

*In particular, the critical surface asymptotes with $\min(\nu_N, \nu_M) = \nu_c$ at low switching ($s \ll \delta$) and with $(\nu_N+\nu_M)/2 = \nu_c$ at high switching ($s \gg \delta$).*

*Proof.* From Theorem 6, the deadly motility regime is implicitly described $0 > r - c_N(s) + d(s)B(s)$. Using $c_N(s) = s + \delta + \nu_N(1 - b_N(s)$, we get the condition in terms of $D(s)$. Notice that $b_N(s)$, $d(s)$ and $B(s)$ are all smooth strictly decreasing functions of $s \in \mathbb{R}^+$, hence $D(s)$ is smooth strictly increasing. The limits follow using the formulae in the Theorem 6. Now, fix $\nu_N, \nu_M$ and vary $s$.

First, we are in the deadly motility regime irrespective of switching $s$ if $D(0) > r - \delta$. Notice that this condition is equivalent to $\nu_N > \nu_c = \frac{\delta(r-\delta)}{2\delta-r}$, where we recognise the critical motility $\nu_c$ in Theorem 1.

Second, we can reach the deadly motility regime for sufficiently high switching rates $s > s_c$, where $s_c$ is the unique critical switching $s_c > 0$ given by $D(s_c) = r - \delta$. Such a critical switching rate exists precisely when $D(\infty) > r - \delta$. This happens precisely when $\frac{\nu_N+\nu_M}{2} > \nu_c = \frac{\delta(r-\delta)}{2\delta-r}$. □

**Theorem 7.** *In the low motility combination $\nu_N, \nu_M \ll \delta$, the non-trivial fixed point of the staircase model is given by:*

$$M_x = \begin{cases} \frac{K}{2}\left(1 - \frac{\delta}{r}\right) + \dots & \text{if } x = 1, \dots, R-1 \\ \frac{K}{2}\left(1 - \frac{\delta+(\nu_N+\nu_M)/2}{r}\right) - \frac{K}{8s}\left(1 - \frac{\delta}{r}\right)(\nu_M - \nu_N) + \dots & \text{if } x = R \\ \frac{K}{4}\left(1 - \frac{\delta}{r}\right)\left(\frac{\nu_N+\nu_M}{\delta} + \frac{\nu_M-\nu_N}{\delta+2s}\right) + + \dots & \text{if } x = R+1 \\ 0 + \dots & \text{if } x = R+2, \dots, L \end{cases}$$
$$N_x = \begin{cases} \frac{K}{2}\left(1 - \frac{\delta}{r}\right) + \dots & \text{if } x = 1, \dots, R-1 \\ \frac{K}{2}\left(1 - \frac{\delta+(\nu_N+\nu_M)/2}{r}\right) - \frac{K}{8s}\left(1 - \frac{\delta}{r}\right)(\nu_N - \nu_M) + \dots & \text{if } x = R \\ \frac{K}{4}\left(1 - \frac{\delta}{r}\right)\left(\frac{\nu_N+\nu_M}{\delta} + \frac{\nu_N-\nu_M}{\delta+2s}\right) + + \dots & \text{if } x = R+1 \\ 0 + \dots & \text{if } x = R+2, \dots, L \end{cases} \tag{S25}$$

*In the high motility combination $\nu_N, \nu_M \gg \delta$, the non-trivial fixed point of the staircase model is given by:*

$$N_x = M_x = \frac{K}{2}\left(1 - \frac{L\delta}{Rr}\right) + \dots. \tag{S26}$$

*In the mixed motility combination $\nu_N \ll \delta \ll \nu_M$, the non-trivial fixed point of the staircase model is given by*

$$\begin{aligned} M_x &= K\left(1 - \frac{\delta + s(1-B(s))}{r}\right)\frac{B(s)}{1+B(s)} + \dots \\ N_x &= \begin{cases} K\left(1 - \frac{\delta+s(1-B(s))}{r}\right)\frac{1}{1+B(s)} + \dots & \text{if } x \le R \\ K\left(1 - \frac{\delta+s(1-B(s))}{r}\right)\frac{sB(s)}{(1+B(s))(s+\delta)} + \dots & \text{if } x > R \end{cases} \end{aligned} \tag{S27}$$

*with*

$$\begin{aligned} A(s) &= \left(\frac{L}{R} - 1\right)\frac{\delta^2 + 2\delta s}{s^2 + \delta s} > 0, \\ B(s) &= \frac{-A + \sqrt{A^2+4}}{2} < 1. \end{aligned} \tag{S28}$$

*Importantly, to lowest order $N_x/M_x = B(s)$ in the region above the staircase $x \ge R$.*

*Proof.* **Low motility combination.** Assume that $\nu_N, \nu_M$ are two independent small parameters, $N_x = a_x + \nu_N b_{Nx} + \nu_M b_{Mx} \dots$, and $M_x = c_x + \nu_M d_{Mx} + \nu_N d_{Nx} \dots$. As before, expand in dimensional powers of $O(\nu_N^k, \nu_M^l)$ to simplify the algebra.

At $O(1)$, the equations describe the local compartmental equilibrium:

$$\begin{aligned} r\left(1 - \frac{a_x + c_x}{K}\right)a_x + sc_x &= (\delta + s)a_x,\ x = 1, \dots, R \\ r\left(1 - \frac{a_x + c_x}{K}\right)c_x + sa_x &= (\delta + s)c_x,\ x = 1, \dots, R \\ + sc_x &= (\delta + s)a_x,\ x = R+1, \dots, L \\ + sa_x &= (\delta + s)c_x,\ x = R+1, \dots, L \end{aligned}$$

Subtracting the first two and the latter two equations, we can see that

$$\begin{aligned} (a_x - c_x)\left[r\left(1 - \frac{a_x + c_x}{K}\right) - 2s - \delta\right] &= 0 \quad, x = 1, \dots, R \\ (a_x - c_x)(2s + \delta) &= 0 \quad, x = R+1, \dots, L \end{aligned}$$

For $x > R$, this implies that $a_x = c_x$, and in turn that $a_x = 0 = c_x$. For $x \le R$, one of the brackets must vanish. If the latter bracket was to vanishes, then the original equations imply that $a_x + c_x = 0$. This is impossible as $a_x, c_x > 0$. Therefore, the first bracket vanishes, $a_x = c_x$ and the original equations imply that

$$a_x = c_x = \frac{K}{2}\left(1 - \frac{\delta}{r}\right).$$

For further use, call this constant $A = \frac{K}{2}\left(1 - \frac{\delta}{r}\right)$. To summarise,

$$\begin{aligned} a_x = c_x &= \frac{K}{2}\left(1 - \frac{\delta}{r}\right),\ x = 1, \dots, R \\ a_x = c_x &= 0,\ x = R+1, \dots, L. \end{aligned}$$

At $O(\nu_N)$, the equations give the first order corrections to curvature as

$$
\begin{aligned}
r\left[\left(1-\frac{3A}{K}\right)b_{Nx}-\frac{A}{K}d_{Nx}\right] \quad & +sd_{Nx}=(\delta+s)b_{Nx}, && x=1,\dots,R-1\\
r\left[\left(1-\frac{3A}{K}\right)d_{Nx}-\frac{A}{K}b_{Nx}\right] \quad & +sb_{Nx}=(\delta+s)d_{Nx}, && x=1,\dots,R-1\\
r\left[\left(1-\frac{3A}{K}\right)b_{Nx}-\frac{A}{K}d_{Nx}\right]-A \quad & +sd_{Nx}=(\delta+s)b_{Nx}, && x=R\\
r\left[\left(1-\frac{3A}{K}\right)d_{Nx}-\frac{A}{K}b_{Nx}\right] \quad & +sb_{Nx}=(\delta+s)d_{Nx}, && x=R\\
+A \quad & +sd_{Nx}=(\delta+s)b_{Nx}, && i=R+1\\
& +sb_{Nx}=(\delta+s)d_{Nx}, && x=R+1\\
& +sd_{Nx}=(\delta+s)b_{Nx}, && x=R+2,\dots,L\\
& +sb_{Nx}=(\delta+s)d_{Nx}, && x=R+2,\dots,L
\end{aligned}
$$

Using the subtraction as before, it can be seen that $b_{Nx}=d_{Nx}=0$ unless $x=R$ or $R+1$. By solving the appropriate system of linear equations for $x=R,R+1$, we also see that

$$
\begin{aligned}
b_{NR} &= -\frac{A}{2}\left(\frac{1}{r-\delta}+\frac{1}{2s}\right)\\
d_{NR} &= -\frac{A}{2}\left(\frac{1}{r-\delta}-\frac{1}{2s}\right)\\
b_{NR+1} &= \frac{A}{2}\left(\frac{1}{\delta}+\frac{1}{\delta+2s}\right)\\
d_{NR+1} &= \frac{A}{2}\left(\frac{1}{\delta}-\frac{1}{\delta+2s}\right)
\end{aligned}
$$

At $O(\nu_M)$, the equations are the same up to swapping $M$ with $N$, and $b$ with $d$.

**High motility combination.** To simplify the algebra, assume a large single parameter $\nu$ such that $\nu_N,\nu_M\sim O(\nu)$, denote the orders $\nu^k$ as $O(k)$ and expand $N_x=a_x+b_x/\nu+\dots$, and $M_x=c_x+d_x/\nu\dots$

At $O(-1)$, the equations describe the motility equilibrium by

$$
\begin{aligned}
a_1 &= a_2,\\
a_{x+1}+a_{x-1} &= 2a_x, && x=1,\dots,L-1\\
a_{L-1} &= a_L,\\
c_1 &= c_2,\\
c_{x+1}+c_{x-1} &= 2c_x, && x=1,\dots,L-1\\
c_{L-1} &= c_L,
\end{aligned}
$$

which imply that $a_x=a$ and $c_x=c$ for some $a,c$ constants.

At $O(0)$, the equations describe the first order correction to curvature due to local compartmental effects by

$$\begin{aligned}
r\left(1-\frac{a+c}{K}\right)a \quad & +\frac{\nu_N}{\nu}b_2+sc &= (\delta+s)a+\frac{\nu_N}{\nu}b_1, & \\
r\left(1-\frac{a+c}{K}\right)a+\frac{\nu_N}{\nu}b_{x-1} \quad & +\frac{\nu_N}{\nu}b_{x+1}+sc &= (\delta+s)a+2\frac{\nu_N}{\nu}b_x, & \quad x=2,\ldots,R \\
+\frac{\nu_N}{\nu}b_{x-1} \quad & +\frac{\nu_N}{\nu}b_{x+1}+sc &= (\delta+s)a+2\frac{\nu_N}{\nu}b_x, & \quad x=R+1,\ldots,L-1 \\
+\frac{\nu_N}{\nu}b_{L-1} \quad & +sc &= (\delta+s)a+\frac{\nu_N}{\nu}b_L, & \\
r\left(1-\frac{a+c}{K}\right)c \quad & +\frac{\nu_M}{\nu}d_2+sa &= (\delta+s)c+\frac{\nu_M}{\nu}d_1, & \\
r\left(1-\frac{a+c}{K}\right)c+\frac{\nu_M}{\nu}d_{x-1} \quad & +\frac{\nu_M}{\nu}d_{x+1}+sa &= (\delta+s)c+2\frac{\nu_M}{\nu}d_x, & \quad x=2,\ldots,R \\
+\frac{\nu_M}{\nu}d_{x-1} \quad & +\frac{\nu_M}{\nu}d_{x+1}+sa &= (\delta+s)c+2\frac{\nu_M}{\nu}d_x, & \quad x=R+1,\ldots,L-1 \\
+\frac{\nu_M}{\nu}d_{L-1} \quad & +sa &= (\delta+s)c+\frac{\nu_M}{\nu}d_L, &
\end{aligned}$$

Summing the first and later four equations, we get

$$\begin{aligned}
Rr\left(1-\frac{a+c}{K}\right)a+Lsc &= L(\delta+s)a, \\
Rr\left(1-\frac{a+c}{K}\right)c+Lsa &= L(\delta+s)c,
\end{aligned}$$

Multiplying the first equation by $c$ and the latter by $a$, and subtracting, we get $Ls(c^2-a^2)=0$. Therefore, $c=a$. Then, we get

$$a=c=\frac{K}{2}\left(1-\frac{L\delta}{Rr}\right).$$

**Mixed motility combination.** Assume that there is a small $\nu$ such that $\nu_N\sim O(\nu)$ and $\nu_M\sim O(1/\nu)$. Then, $N_x=a_x+b_x\nu+\ldots$, and $M_x=c_x+d_x\nu\ldots$.

At $O(-1)$, the equations for high motility equilibrium are:

$$\begin{aligned}
c_1 &= c_2, \\
c_{x+1}+c_{x-1} &= 2c_x, \quad x=1,\ldots,L-1 \\
c_{L-1} &= c_L.
\end{aligned}$$

This result implies that there is a constant $c$ such that $c_x=c$.

At $O(0)$, the local dynamics of the slow population involves the faster population:

$$\begin{aligned}
r\left(1-\frac{a_x+c}{K}\right)a_x \quad & +sc &= (\delta+s)a_x, & \quad x=1,\ldots,R \\
& +sc &= (\delta+s)a_x, & \quad x=R+1,\ldots,L \\
r\left(1-\frac{a_1+c}{K}\right)c \quad & +\nu_M\nu d_2+sa_1 &= (\delta+s)c+\nu_M\nu d_1, & \\
r\left(1-\frac{a_x+c}{K}\right)c+\nu_M\nu d_{x-1} \quad & +\nu_M\nu d_{x+1}+sa_x &= (\delta+s)c+2\nu_M\nu d_x, & \quad x=2,\ldots,R \\
+\nu_M\nu d_{x-1} \quad & +\nu_M\nu d_{x+1}+sa_x &= (\delta+s)c+2\nu_M\nu d_x, & \quad x=R+1,\ldots,L-1 \\
+\nu_M\nu d_{L-1} \quad & +sa_L &= (\delta+s)c+\nu_M\nu d_L, &
\end{aligned}$$

Notice that the first equation can be thought of as a quadratic equation for $a_x$ when $c$ is thought of as a parameter:

$$0=f(a_x)=-\frac{r}{K}a_x^2+\left[\left(1-\frac{c}{K}\right)-(\delta+s)\right]a_x+sc.$$

Since $-r/K < 0$ and $f(0) = sc > 0$, the parabola has exactly one root with $a_x > 0$. Therefore, $a_x = a$ when $x \le R$. The second equation similarly gives that $a_x = b$ when $x > R$.

By summing the other equations, we get the system for variables $a, b, c$:

$$r\left(1 - \frac{a+c}{K}\right)a + sc = (\delta + s)a, \tag{S36}$$

$$+ sc = (\delta + s)b, \tag{S37}$$

$$Rr\left(1 - \frac{a+c}{K}\right)c + s(Ra + (L-R)b) = L(\delta + s)c. \tag{S38}$$

Firstly, eliminate $b$ from the equation (S38) to get

$$Rr\left(1 - \frac{a+c}{K}\right)c + Rsa + (L-R)\frac{s^2}{\delta + s}c = L(\delta + s)c \tag{S39}$$

Multiplying equation (S39) by $a$ and subtracting it from the equation (S36) multiplied by $Rc$, we can obtain

$$\frac{a}{c} - \frac{c}{a} = \left(\frac{L}{R} - 1\right)\frac{\delta^2 + 2\delta s}{s^2 + \delta s} := A(s), \tag{S40}$$

which defines a dimensionless number $A(s) > 0$. The equation for $c/a$ can be solved by requiring that $c/a > 0$:

$$\frac{c}{a} = \frac{-A + \sqrt{A^2 + 4}}{2} := B(s) < 1, \tag{S41}$$

where $B(s)$ is the proportion of high motility phenotype to low motility phenotype above the staircase. Combining equations (S36) and (S41), it is straightforward to find the solution

$$\begin{aligned} a &= K\left(1 - \frac{\delta + s(1 - B(s))}{r}\right)\frac{1}{1 + B(s)}, \\ b &= K\left(1 - \frac{\delta + s(1 - B(s))}{r}\right)\frac{sB(s)}{(1 + B(s))(s + \delta)}, \\ c &= K\left(1 - \frac{\delta + s(1 - B(s))}{r}\right)\frac{B(s)}{1 + B(s)}. \end{aligned} \tag{S42}$$

□

**Theorem 8.** *At low switching $s \ll \delta$, the overall wild-type profile behaves as if it has a single motility of $\nu = \min(\nu_N, \nu_M)$. At high switching $s \gg \delta$, the overall wild-type profile behaves as if it has a single motility of $\nu = (\nu_N + \nu_M)/2$.*

*In particular, this result affects the deadly motility regime in the $(\nu_N, \nu_M, s)$ space. Let $\overline{f} = (rR - \delta L)/L$ be the average wild-type fitness. The wild-type profile is non-trivial iff:*

- *the environment is source-like $\overline{f} > 0$, motility rates $\nu_N, \nu_M$ are arbitrary, and switching rate $s$ is arbitrary,*
- *the environment is sink-like $\overline{f} < 0$ and*
  - *switching is low $s \ll \delta$ and the slower phenotype is in the deadly motility regime $\min(\nu_N, \nu_M) > \nu_c$,*
  - *switching is high $s \gg \delta$ and the average-motility phenotype is in the deadly motility regime $(\nu_N + \nu_M)/2 > \nu_c$.*

*Proof.* As for the source-sink model, the deadly motility regime corresponds to region in the $(\nu_N, \nu_M, s)$ space. Now, we will fix the motility rates $\nu_N, \nu_M$ and vary $s$.

At high switching ($s \gg \delta$), the dominant dynamics $\dot{N}_x \approx (M_x - N_x)$ (and $N_x \leftrightarrow M_x$) pushes the dynamics close to the surface $N_x = M_x$. On this surface, we can define coordinates $N'_x = N_x + M_x$ and derive the dynamics

$$\begin{aligned} \dot{N}'_1 &= r\left(1 - \frac{N'_1}{K}\right)N'_1 \quad -\delta N'_1 + \frac{\nu_N + \nu_M}{2}(N'_2 - N'_1), \\ \dot{N}'_x &= r\left(1 - \frac{N'_x}{K}\right)N'_x \quad -\delta N'_x + \frac{\nu_N + \nu_M}{2}(N'_{x-1} + N'_{x+1} - 2N'_x),\ x = 2, \dots, R \\ \dot{N}'_x &= \quad -\delta N'_x + \frac{\nu_N + \nu_M}{2}(N'_{x-1} + N'_{x+1} - 2N'_x),\ x = R+1, \dots, L-1 \\ \dot{N}'_L &= \quad -\delta N'_L + \frac{\nu_N + \nu_M}{2}(N'_{L-1} - N'_L), \end{aligned}$$

Therefore, the overall population $N'_x$ behaves as a population with average motility. In particular, the overall population is in the deadly motility regime at high switching ($s \gg \delta$) iff the average-motility population is.

For low switching, we prove the stability of the non-trivial fixed point at $s = 0$. This fixed point is given by $M_x = 0$ and $N_x$, which coincides with the non-trivial fixed point of the staircase model for a single phenotype of motility $\nu = \nu_N$, i.e., for the system with equation (S6). The corresponding Jacobian can be derived from equation (S16) and has the form

$$J = \begin{pmatrix} J_1 & \star \\ 0 & J_2(\nu_M) \end{pmatrix},$$

where

$$J_1 = \begin{pmatrix} r(1-2N_1/K) - \nu_N - \delta & \nu_N & 0 & \dots \\ \nu_N & r(1-2N_2/K) - 2\nu_N - \delta & \nu_N & \dots \\ 0 & \nu_N & r(1-2N_3/K) - 2\nu_N - \delta & \dots \\ \vdots & \vdots & \vdots & \ddots \end{pmatrix}$$

and

$$J_2(\nu_M) = \begin{pmatrix} r(1-N_1/K) - \nu_M - \delta & \nu_M & 0 & \dots \\ \nu_M & r(1-N_2/K) - 2\nu_M - \delta & \nu_M & \dots \\ 0 & \nu_M & r(1-N_3/K) - 2\nu_M - \delta & \dots \\ \vdots & \vdots & \vdots & \ddots \end{pmatrix}$$

We prove the stability of the non-trivial fixed point if we show that the eigenvalues of $J$ have negative real part. Notice that the set of eigenvalues of $J$ is the union of the sets of eigenvalues of $J_1$ and $J_2(\nu_M)$. Therefore, it suffices to show that the eigenvalues of $J_1$ and $J_2(\nu_M)$ are negative. The eigenvalues of $J_1$ must be negative since $J_1$ is the Jacobian corresponding to the non-trivial fixed point of the staircase model for a single phenotype with motility $\nu_N$, which is stable from previous analysis. The proof that the eigenvalues of $J_2(\nu_M)$ are negative for $\nu_M > \nu_N$ is identical to the proof Hastings,[S27] which consists of two steps. First, one shows that the largest eigenvalue of $J_2(\nu_N)$ is 0 because $(N_1, \dots, N_L)$ is the eigenvector and only the largest eigenvalue can have an eigenvector with positive entries. Second, one shows that this eigenvalue increases as $\nu_M$ increases from $\nu_N$. To sum up, the overall population is dominated by the slower population $N_x$ at low switching ($s \ll \delta$).

The result for the existence of the deadly motility regime follows, as the system behaves as a single phenotype of single motility which interpolates between the slower motility $\min(\nu_N, \nu_M)$ (at low switching $s \ll \delta$) and the average motility $(\nu_N + \nu_M)/2$ (at high switching $s \gg \delta$). □

## Supplementary Note 10. Figure Notes.

This Supplementary Note provides the parameter values used for producing all our figures as well as other specific guidance for reproducing our results fully. Unless stated otherwise, we follow previous work[S7] and use as lattice size $L = 8$, carrying capacity $K = 10^5$, mutation rates $\mu_f = 10^{-7}h^{-1}$ and $\mu_b = 10^{-4}h^{-1}$, division rate $r = 1h^{-1}$ and death rate $\delta = 0.1h^{-1}$ (resp. $\delta = 0.3h^{-1}$) for source-like (resp. sink-like) environments.

Specifically, in Fig. 1, the death rate is $\delta = 0.1$. Panel b was created from a simulation of the staircase model with $\nu = 10^{-2}h^{-1}$ for low motility and $\nu = 1h^{-1}$ for high motility. Panel d was created from the same simulations. Moreover, the black curve describing the total population numbers was plotted by finding the non-trivial fixed point of the mean-field theory in equation (S6) via the Newton-Raphson method.

In Fig. 2, the motility rate $\nu$ is varied in logarithmic steps between $10^{-5}h^{-1}$ and $10h^{-1}$, and the appropriate death rate $\delta = 0.1h^{-1}$, $0.3h^{-1}$ is chosen for the source-like and sink-like environments, respectively. The dots of panel b correspond to simulations while the lines correspond to the analytical techniques. Panel c was produced by the analytical techniques. Panel d was plotted by finding the non-trivial fixed point of the mean-field theory in equation (S6) via the Newton-Raphson method.

In Fig. 3, the death rate is $\delta = 0.3h^{-1}$. In panel b, the switching rates $s$ are $s = 0h^{-1}$ (low switching $s \ll \delta$) and $s = 5h^{-1}$ (high switching $s \gg \delta$), motility rates $\nu_{1,2}$ are varied in logarithmic steps between $10^{-5}h^{-1}$ and $10h^{-1}$

and the resulting heatmap plots of the adaptation rate are constructed for resistance state $R = 5$ (Fig. 1b) from simulations, where $R$ is the genotype of highest resistance before the adaptation jump $R \to R+1$. In panel c, the switching rates $s$ are $s = 10^{-3}h^{-1}$ (low switching $s \ll \delta$) and $s = 5h^{-1}$ (high switching $s \gg \delta$), motility combinations are represented by $(\nu_1, \nu_2) = (10^{-2}h^{-1}, 10^{-4}h^{-1})$ (low motility), $(\nu_1, \nu_2) = (3.16h^{-1}, 10^{-4}h^{-1})$ (mixed motility) and $(\nu_1, \nu_2) = (3.16h^{-1}, 1h^{-1})$ (high motility). Wild-type profiles are plotted for resistance state $R = 4$ from simulations. Black curves describe the total number of cells in each phenotype and were plotted by finding the non-trivial fixed point of the mean-field theory with equation (S43) via the Newton-Raphson method,

$$\begin{aligned} 0 &= \quad -sN_x + sM_x + r\left(1 - \frac{N_x + M_x}{K}\right) N_x \mathbb{1}_{x \le R} - \delta N_x + \nu(N_{x-1} - N_x)\mathbb{1}_{1<x} + \nu(N_{x+1} - N_x)\mathbb{1}_{x<L} \\ 0 &= \ +sN_x - sM_x + r\left(1 - \frac{N_x + M_x}{K}\right) M_x \mathbb{1}_{x \le R} - \delta M_x + \nu(M_{x-1} - M_x)\mathbb{1}_{1<x} + \nu(M_{x+1} - M_x)\mathbb{1}_{x<L} \end{aligned} \tag{S43}$$

In panel b of Fig. 4, the death rate is $\delta = 0.3h^{-1}$ and switching thresholds $S$ are $S = 9.5 \times 10^4$ (high threshold $S \gg K(1 - \delta/r)$) and $S = 100$ (low threshold $S \ll K(1 - \delta/r)$). Motility rates $\nu_{L,H}$ are varied in logarithmic steps between $10^{-5}h^{-1}$ and $10h^{-1}$ and the resulting heatmap plots of the adaptation rate are constructed for resistance state $R = 2$ from simulations. In panel c, the death rate is $\delta = 0.1h^{-1}$ and switching thresholds $S$ are $S = 9.5 \times 10^4$ (high threshold $S \gg K(1 - \delta/r)$) and $S = 10^{4.5}$ (low threshold $S \ll K(1 - \delta/r)$). This choice is different from the one used in panel b but allows for better visualisation of the wild-type profiles when the scale of cell numbers is kept linear as in previous figures. The mixed motility combination is represented by $(\nu_L, \nu_H) = (3.14h^{-1}, 10^{-4}h^{-1})$ (fast-to-slow) and $(\nu_L, \nu_H) = (10^{-4}h^{-1}, 3.14h^{-1})$ (slow-to-fast). The wild-type profiles (including the black curves) are plotted for resistance state $R = 1$ from simulations. The Newton-Raphson method is not reliable in this case because the step-function used for density-dependent motility is not smooth.

In Fig. 5, we consider $L = 2$ and $R = 1$. $K = 10^5$ and $r = 1h^{-1}$ are chosen as usual and mutation rates do not to be chosen as they do not affect the dynamics on ecological time-scales. In panel b, the phase portraits are plotted with matplotlib[S28] for the choice of the death rate $\delta = 0.25h^{-1}$ and the motility rates $\nu = 0h^{-1}$, $0.1h^{-1}$, $100h^{-1}$ (source-like environment), resp. for the death rate $\delta = 0.75h^{-1}$ and the motility rates $\nu = 0h^{-1}$, $0.1h^{-1}$, $5h^{-1}$ (sink-like environment). In panel c, the bifurcation diagrams are plotted using the solution for the non-trivial fixed point in (S1). The source-like environment is represented by $\delta = 0.25h^{-1}$ and the sink-like environment is represented by $\delta = 0.75h^{-1}$.

In Supplementary Fig. 1, the motility rate $\nu$ is varied in logarithmic steps between $10^{-5}h^{-1}$ and $10h^{-1}$, and the appropriate death rate $\delta = 0.1h^{-1}$, $0.3h^{-1}$ is chosen for the source-like and sink-like environments, respectively. The dots of panel b correspond to simulations while the lines correspond to the analytical techniques.

In Supplementary Fig. 2, the death rate $\delta = 0.1h^{-1}$, $0.3h^{-1}$ is chosen for source-like and sink-like environment, respectively. Motility rate $\nu$ is varied in logarithmic steps between $10^{-5}h^{-1}$ and $10h^{-1}$ and resistance cost $c$ is varied in logarithmic steps between $10^{-4.5}h^{-1}$ and $10^{-0.5}h^{-1}$. The resulting heatmap plots of adaptation rate are constructed for resistance state $R = 3$ from simulations.

In Supplementary Fig. 3, the death rate $\delta = 0.1h^{-1}$, $0.3h^{-1}$ is chosen for source-like and sink-like environment, respectively. Motility rate $\nu$ is varied in logarithmic steps between $10^{-5}h^{-1}$ and $10h^{-1}$ and mutation rate $\mu_f$ is varied in logarithmic steps between $10^{-7}h^{-1}$ and $10^{-3}h^{-1}$. The resulting heatmap plots of adaptation rate are constructed for resistance state $R = 4$ from simulations.

In Supplementary Fig. 4, the death rate $\delta = 0.1h^{-1}$, $0.3h^{-1}$ is chosen for source-like and sink-like environment, respectively. Motility rate $\nu$ is varied in logarithmic steps between $10^{-5}h^{-1}$ and $10h^{-1}$ and competition strength $\alpha$ is varied in linear steps between 0 and 1. The resulting heatmap plots of adaptation rate are constructed for resistance state $R = 5$ from simulations.

In Supplementary Fig. 5, the death rate is $\delta = 0.1h^{-1}$. Motility rate $\nu$ is varied in logarithmic steps between $10^{-5}h^{-1}$ and $10h^{-1}$ and the multiplicative increase in death rate $\sigma$ is varied linearly between 1 and 9. The resulting heatmap plots of the adaptation rate are constructed for resistance state $R = 5$ from simulations.

In Supplementary Fig. 6, the death rate is $\delta = 0.1h^{-1}$. Motility rate $\nu$ is varied in logarithmic steps between $10^{-5}h^{-1}$ and $10h^{-1}$ and the chemotactic probability to move up the antibiotic gradient $p$ is varied linearly between 0.1 and 0.9. The resulting heatmap plots of the adaptation rate are constructed for resistance state $R = 5$ from simulations.

In panel a of Supplementary Fig. 7 the switching rate is varied in logarithmic steps between $10^{-5}h^{-1}$ and $10h^{-1}$. The mixed motility combination is chosen as $(\nu_1, \nu_2) = (10^{-3}, 10)$ and the appropriate death rate $\delta = 0.1h^{-1}$, $0.3h^{-1}$ is chosen for the source-like and sink-like environment, respectively. The dots of panel b correspond to simulations while the lines are obtained by analytical techniques for a population of homogeneous motility $\min(\nu_1, \nu_2)$ at low switching and $(\nu_1 + \nu_2)/2$ at high switching. In panel b, the death rate is $\delta = 0.1h^{-1}$ and the switching rate is varied $s = 0h^{-1}$, $0.001h^{-1}$, $0.01h^{-1}$, $0.1h^{-1}$, $1h^{-1}$. Motility rates $\nu_{1,2}$ are varied in logarithmic steps between $10^{-5}h^{-1}$

and $10h^{-1}$ and the resulting heatmap plots of the adaptation rate are constructed for resistance state $R = 5$ from simulations. In panel c, the death rate is $\delta = 0.3h^{-1}$ and the switching rate is varied $s = 0.1h^{-1}$, $0.4h^{-1}$, $0.67h^{-1}$, $0.9h^{-1}$, $1h^{-1}$, $10h^{-1}$. Motility rates $\nu_{1,2}$ are varied in logarithmic steps between $\nu = 10^{-5}h^{-1}$ and $\nu = 10h^{-1}$. The survival probability is computed as follows. We run the Newton-Raphson method to search for a fixed point of equation (S43) in the non-negative quadrant $N_x, M_x \geq 0$. If the search returns the trivial (resp. non-trivial) fixed point, we set the survival probability to 0 (resp. 1).

Supplementary Fig. 8b reproduces the data of Fig. 4b. The data is reproduced to facilitate comparisons between the implicit and explicit models. In panels c, d and e of Supplementary Fig. 8, the switching thresholds $S$ are $S = 9.5 \times 10^4$ (high threshold $S \gg K(1 - \delta/r)$) and $S = 100$ (low threshold $S \ll K(1 - \delta/r)$). Motility rates $\nu_{L,H}$ are varied in logarithmic steps between $10^{-5}h^{-1}$ and $10h^{-1}$ and the resulting heatmap plots of the adaptation rate are constructed for resistance state $R = 2$ from simulations. Furthermore, in panel c, the death rate is $\delta = 0.1h^{-1}$. In panel d, the death rate is $\delta = 0.3h^{-1}$, the switching rate is $s = 1$ and the switching preference is $\beta = 10^{-3}$. In panel e, the death rate is $\delta = 0.3h^{-1}$, the switching rate is $s = 1$ and the switching preference is $\beta = 10^{-3}$. In panel f, the death rate is $\delta = 0.3h^{-1}$, the switching threshold is $S = 5 \times 10^4$, the switching rate is $s = 10$, and the switching preference is $\beta = 10^{-3}$. For slow-to-fast switching, the motility rates are $\nu_L = 10^{-3}h^{-1}$, $\nu_H = 10^{0.5}h^{-1}$. For fast-to-slow switching, the motility rates are $\nu_L = 10^{0.5}h^{-1}$, $\nu_H = 10^{-3}h^{-1}$. Snapshots are taken at time $t = 1000h$.

In Supplementary Fig. 9, the death rate $\delta = 0.1h^{-1}$, $0.3h^{-1}$ is chosen for the source-like and sink-like environment, respectively. Motility rate $\nu$ is varied in logarithmic steps between $10^{-5}h^{-1}$ and $10h^{-1}$ and the horizontal gene transfer rate $h$ is in logarithmic steps between $10^{-7}h^{-1}$ and $10^{-3}h^{-1}$. The resulting heatmap plots of the adaptation rate are constructed for resistance state $R = 4$ from simulations.

## SUPPLEMENTARY FIGURES

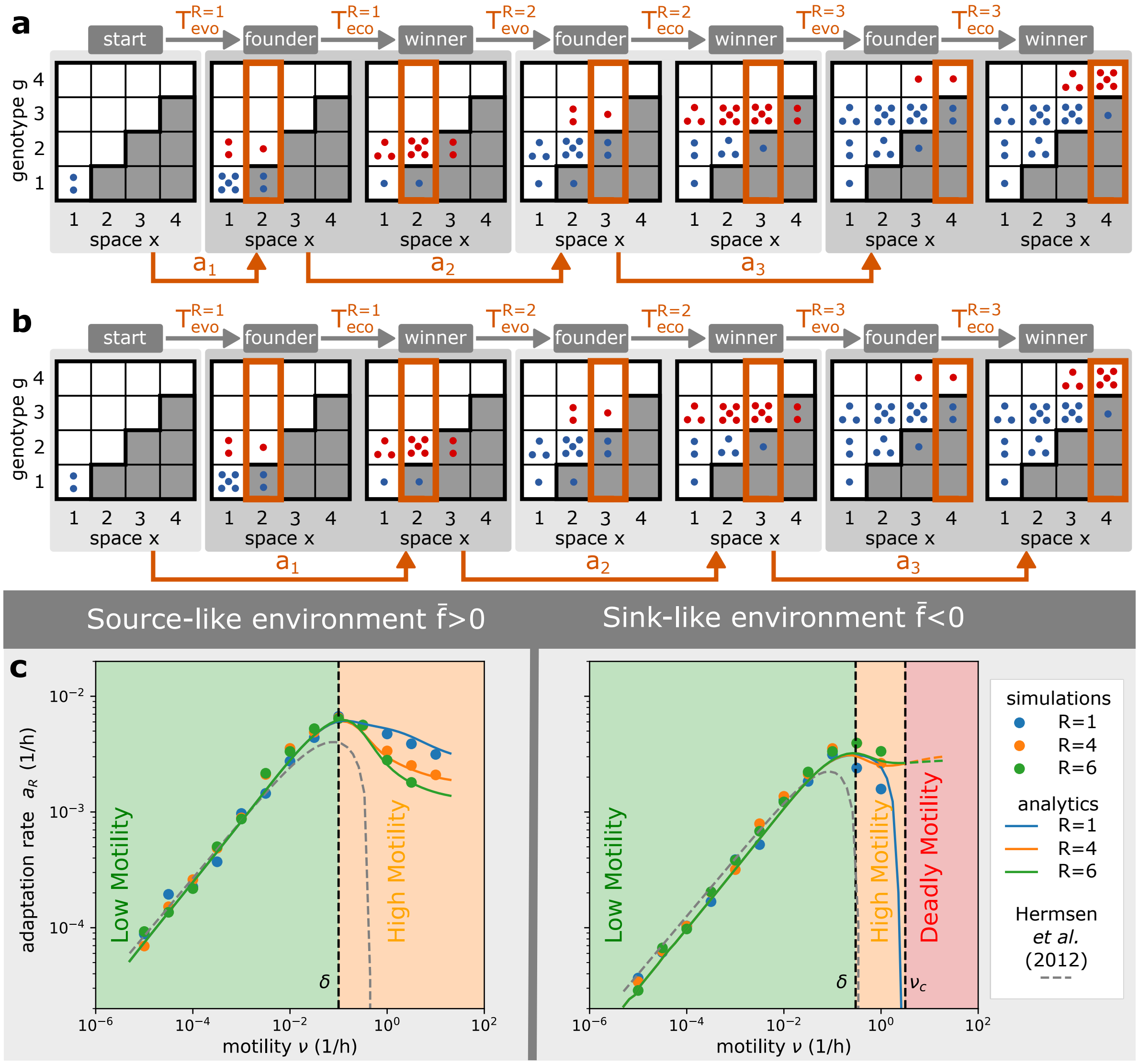


**Supplementary Figure** 1 | **Alternative definition of adaptation rate. (a)** Adaptation rate between consecutive founder states (i.e., a first mutant appears in the overlap region). Same terminology as in Fig. 2a. The adaptation rate is defined as the rate at which consecutive founder states appear. This definition is adapted from Hermsen *et al.*[S7] and used throughout our work. **(b)** Adaptation rate between consecutive winner states (i.e., mutants outgrow wild-type in the overlap region). The adaptation rate is defined as the rate at which consecutive winner states appear. **(c)** Adaptation rate as a function of motility rate. Same description as in Fig. 2b, but the definition of adaptation rate used rests on winner states. These results show that our conclusions hold for both definitions of adaptation rate. Parameters: $L = 8$, $K = 10^5$, $r = 1/h$, $\delta = 0.1/h$ (left), $\delta = 0.3/h$ (right), $\mu_b = 10^{-4}/h$, $\mu_f = 10^{-7}/h$.

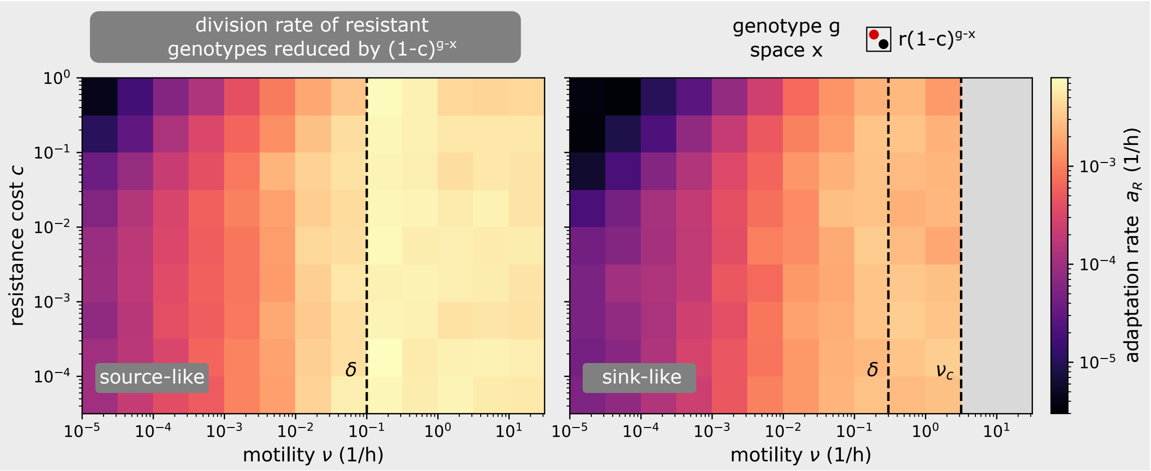


**Supplementary Figure** 2 | **Effect of resistance costs.** Resistance cost $c$ is modelled as a reduction in the division rate of genotypes $g$ at position $x$ by a factor of $(1-c)^{g-x}$ as in Hermsen *et al.*.[S7] The heatmap of adaptation rate on the $(\nu, c)$ plane explains how resistance cost $c$ changes the relationship between motility rate $\nu$ and adaptation rate $a_R$. Generically, resistance cost does not affect the relationship between adaptation rate and motility: in the low motility regime, motility increases bacterial adaptation, but the inverse occurs in the high motility regime. Only when the motility rate is very low $\nu \ll \delta$ and resistance cost is very high $c > \sqrt{\nu/\delta}$, the adaptation rate is decreased by $c$, as proved in Hermsen *et al.*.[S7] However, such resistance costs are considered high.[S8] Even if the motility is low as $\nu = 10^{-2}\delta$ (i.e., 99% of bacteria do not migrate during their lifetime), the resistance cost is important only if $c > 0.1$.[S8] Moreover, resistance cost cannot influence the deadly motility regime (grey) as in this regime the susceptible wild-type goes extinct. A detailed description of this model can be found in Supplementary Note 4. Parameters: $L = 8$, $K = 10^5$, $r = 1/h$, $\delta = 0.1/h$ (left), $\delta = 0.3/h$ (right), $\mu_b = 10^{-4}/h$, $\mu_f = 10^{-7}/h$.

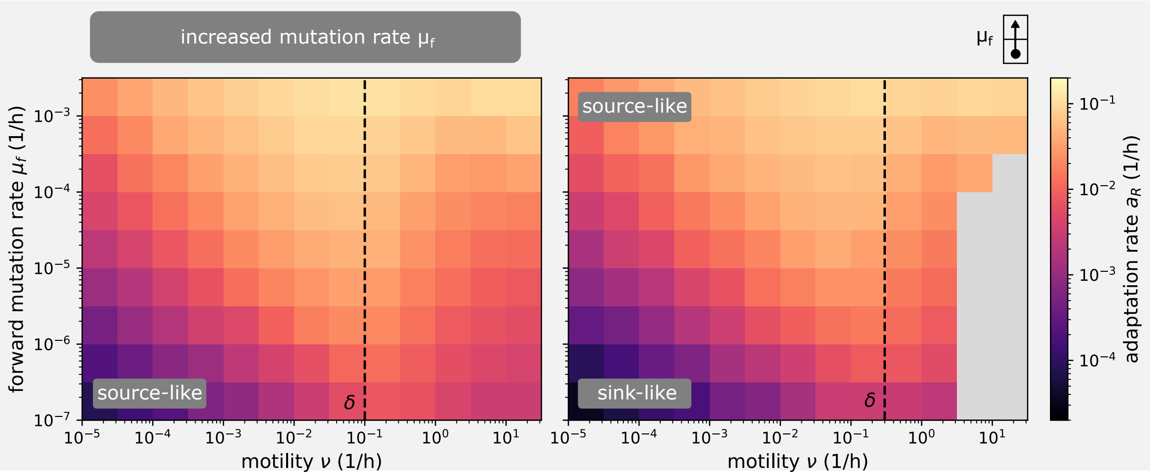


**Supplementary Figure** 3 | **Effect of mutation rate.** Here, the forward (resistance-conferring) mutation rate $\mu_f$ is varied. The heatmap of adaptation rate on the $(\nu, \mu_f)$ plane explains how the mutation rate $\mu_f$ changes the relationship between motility rate $\nu$ and adaptation rate $a_R$. Mutation rate below $\mu_f < 10^{-4}$ does not affect the relationship between adaptation rate and motility: adaptation rate increases at low motility, decreases at high motility, and no adaptation occurs at deadly motility (grey). This mutation rate threshold corresponds to the probability of $\mu_f/\delta = 10^{-3}$ resistance mutations per cell division, which is very high compared to experimental estimates of $10^{-6}$ and $10^{-9}$ resistance mutations per cell division.[S9–S11] A detailed description of this model can be found in Supplementary Note 4. Parameters: $L = 8$, $K = 10^5$, $r = 1/h$, $\delta = 0.1/h$ (left), $\delta = 0.3/h$ (right), $\mu_b = 10^{-4}/h$, $\mu_f = 10^{-7}/h$.

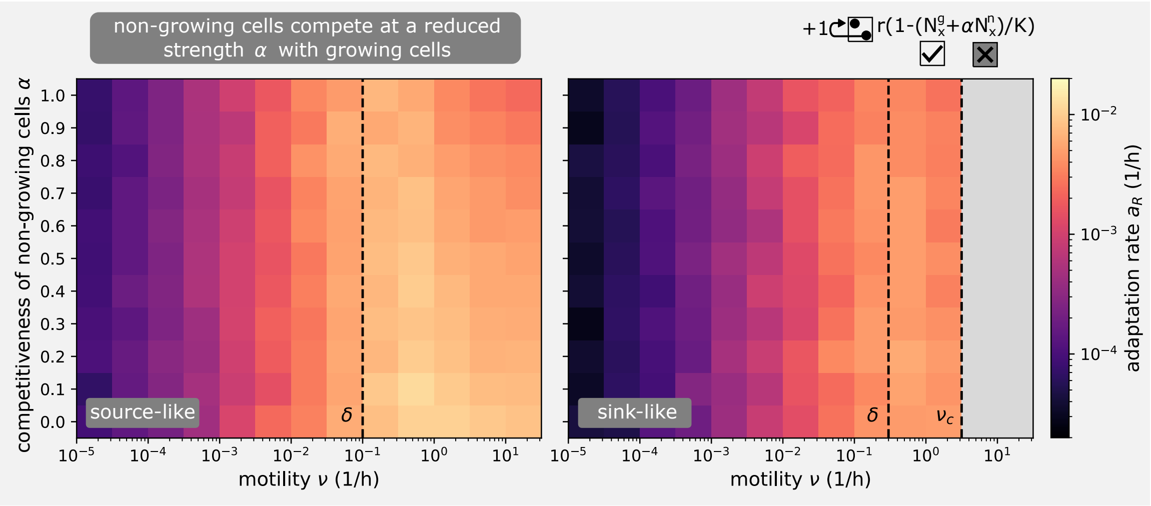


**Supplementary Figure** 4 | **Differential competition between growing and non-growing cells.** In the main text, growing and non-growing cells contribute to the carrying capacity in the same way, even though non-growing cells can be weaker competitors. To understand the effect of such differential competition, we now consider that non-growing cells contribute to the carrying capacity at a reduced rate $\alpha \in [0, 1]$ compared to growing cells ($\alpha = 0$ corresponds to no contribution, $\alpha = 1$ corresponds to the same contribution as in the original model). Therefore, the division rate of growing cells ($g \geq x$) is $\max(0, r(1 - (N_x^g + \alpha N_x^n)/K)$, where $N_x^g$ is the number of growing cells above the staircase ($g \geq x$) and $N_x^n$ is the number of non-growing cells below the staircase ($g < x$) in a given spatial compartment $x$. The heatmap of adaptation rate on the $(\nu, \alpha)$ plane explains how the reduction in competitive strength of non-growing cells $\alpha$ changes the relationship between motility rate $\nu$ and adaptation rate $a_R$. Importantly, there is no significant change in the relationship between adaptation rate and motility: adaptation rate increases at low motility, decreases at high motility, and no adaptation occurs at deadly motility (grey). A detailed description of this model can be found in Supplementary Note 4. Parameters: $L = 8$, $K = 10^5$, $r = 1/h$, $\delta = 0.1/h$ (left), $\delta = 0.3/h$ (right), $\mu_b = 10^{-4}/h$, $\mu_f = 10^{-7}/h$.

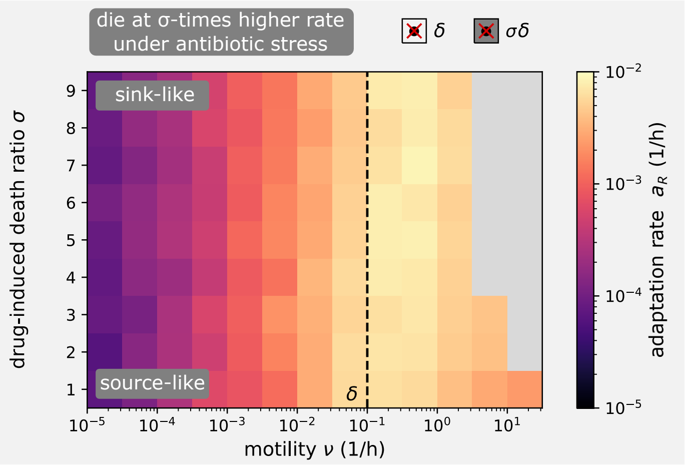


**Supplementary Figure** 5 | **Effect of bactericidal antibiotics.** Bactericidal antibiotics are considered by increasing the death rate of susceptible genotypes $g < x$ (under the staircase) $\sigma$-times. The heatmap of adaptation rate on the $(\nu, \sigma)$ plane explains how $\sigma$ changes the relationship between motility $\nu$ and adaptation rate $a_R$. In the low motility regime $\nu < \delta$, the adaptation rate increases with motility $\nu$ and is unaffected by $\sigma$. In the high motility regime $\nu > \delta$, the adaptation rate decreases with motility $\nu$. Moreover, even if the environment is source-like for $\sigma = 1$, the high motility regime can transition into the deadly motility regime (grey) provided the bactericidal effect is strong enough $\sigma \gg 1$. A detailed description of this model can be found in Supplementary Note 4. Parameters: $L = 8$, $K = 10^5$, $r = 1/h$, $\delta = 0.1/h$, $\mu_b = 10^{-4}/h$, $\mu_f = 10^{-7}/h$.

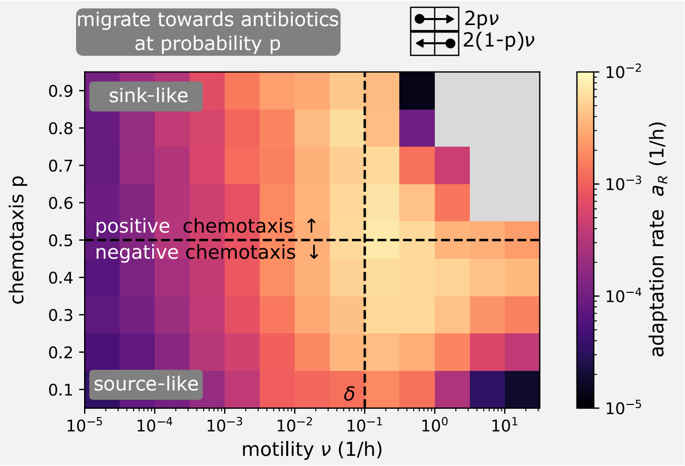


**Supplementary Figure** 6 | **Effect of chemotaxis.** Motility bias in the chemical gradient (chemotaxis) is considered by varying the probability $p$ that a cell moves up or down the antibiotic gradient. In our original model, cell motility is assumed random, $p = 0.5$. Cell movement is biased up the antibiotic gradient if $p > 0.5$ (positive chemotaxis), and down the gradient $p < 0.5$ (negative chemotaxis). The heatmap of adaptation rate on the $(\nu, \sigma)$ plane explains how $p$ changes the relationship between motility $\nu$ and adaptation rate $a_R$. In the low motility regime $\nu < \delta$, the adaptation rate increases with motility $\nu$ and is unaffected by chemotaxis $p$. In high motility regime $\nu > \delta$, positive chemotaxis $p > 0.5$ converts the high motility regime into the deadly motility regime (grey) even when the environment is source-like at $p = 0.5$. Negative chemotaxis $p < 0.5$ prevents the deadly motility regime and slows down the adaptation in the high motility regime, compared to unbiased motion $p = 0.5$. A detailed description of this model can be found in Supplementary Note 4. Parameters: $L = 8$, $K = 10^5$, $r = 1/h$, $\delta = 0.1/h$, $\mu_b = 10^{-4}/h$, $\mu_f = 10^{-7}/h$.

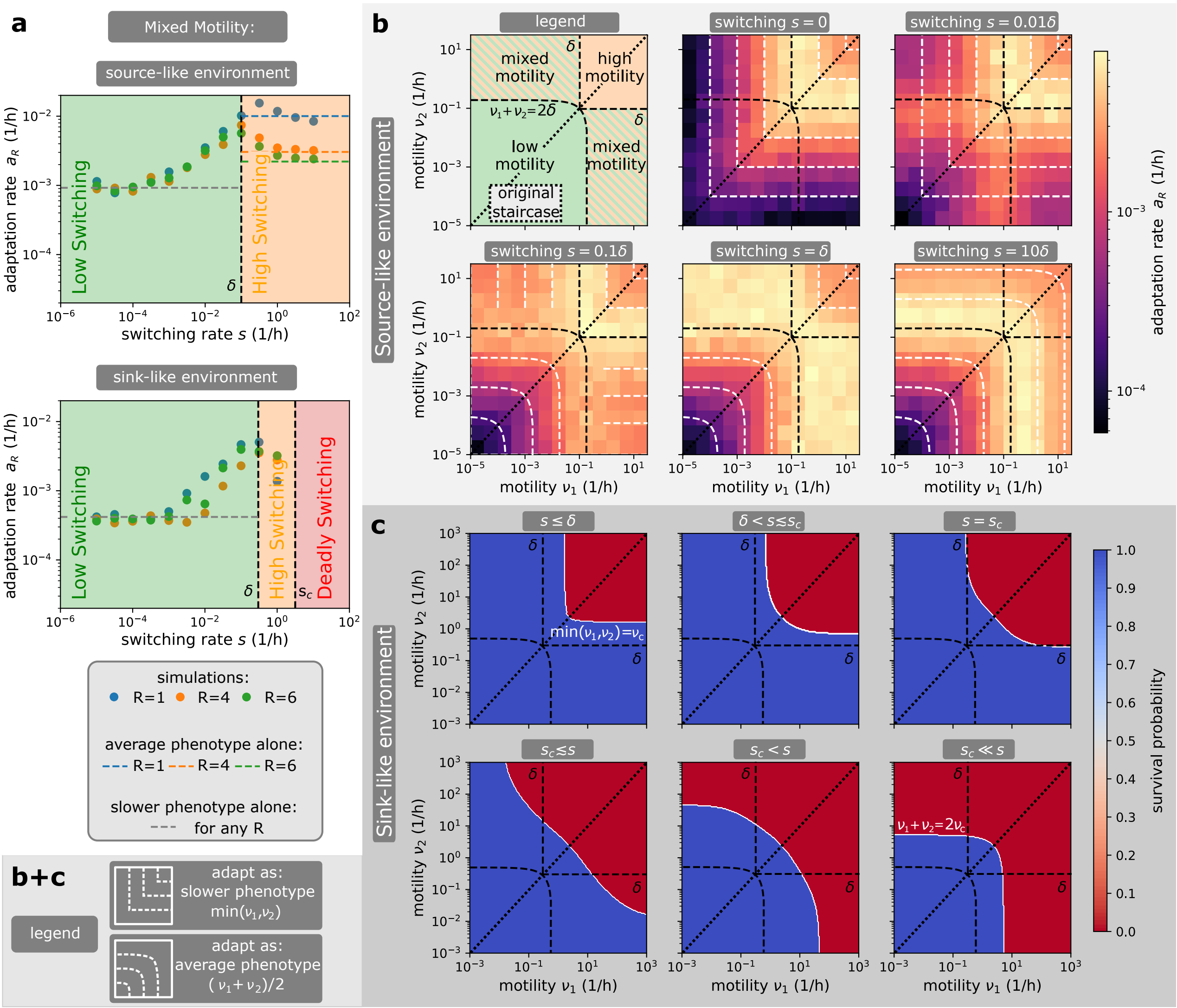


**Supplementary Figure 7 | Further effects of switching between motility phenotypes stochastically. (a)** Switching rate $s$ modifies the adaptation rate $a_R$ and the adaptation regime in the mixed motility combination $\nu_1 \ll \delta \ll \nu_2$. Dots correspond to simulations, while the asymptotic lines are derived with analytical techniques. At low switching ($s \ll \delta$), the population adapts as if a single slower phenotype is present, whose adaptation rate is independent of the resistance state $R$ (grey dashed line). At high switching ($s \ll \delta$), the population behaves as if a single phenotype of average motility is present, whose adaptation rate depends on the number of compartments $R$ where wild-type can divide (coloured dashed lines). Moreover, if the environment is sink-like and the average motility is above the critical motility, there is a critical switching rate $s_c$ which limits bacterial survival. **(b)** Adaptation rate heatmap on the $(\nu_1, \nu_2)$ plane for different switching rates $s$. This is the same plot as in Fig. 3b but for a source-like environment instead of a sink-like environment. The $(\nu_1, \nu_2)$ plane can be partitioned into different combinations of adaptation regimes and its diagonal corresponds to a population of a single motility. Bacteria adapt as if all cells had the same effective motility, which corresponds to the intersection of the level sets (white dashed lines) with the diagonal. At low switching rate $s$, the effective motility matches the slower motility phenotype present $\min(\nu_1, \nu_2)$. At high switching rate $s$, the effective motility matches the average motility $(\nu_1 + \nu_2)/2$. Unlike for the sink-like environment, there is no deadly motility regime for any motility $\nu_{1,2}$ and switching $s$ in the source-like environment. **(c)** Deadly motility regime and critical surface. In sink-like environments, there is an abrupt change in survival probability from 1 to 0 when the population is sufficiently well-mixed. The deadly motility regime corresponds to the space with zero survival probability (red) and is bounded by a critical surface (white). The critical surface asymptotes with $\min(\nu_1, \nu_2) = \nu_c$ at low switching ($s \ll \delta$) and with $(\nu_1 + \nu_2)/2 = \nu_c$ at high switching ($s \gg \delta$). Find a detailed description of this model in Supplementary Note 5. Parameters: $L = 8$, $K = 10^5$, $r = 1/h$, $\delta = 0.1/h$ (source-like environment), $\delta = 0.3/h$ (sink-like environment), $\mu_b = 10^{-4}/h$, $\mu_f = 10^{-7}/h$.

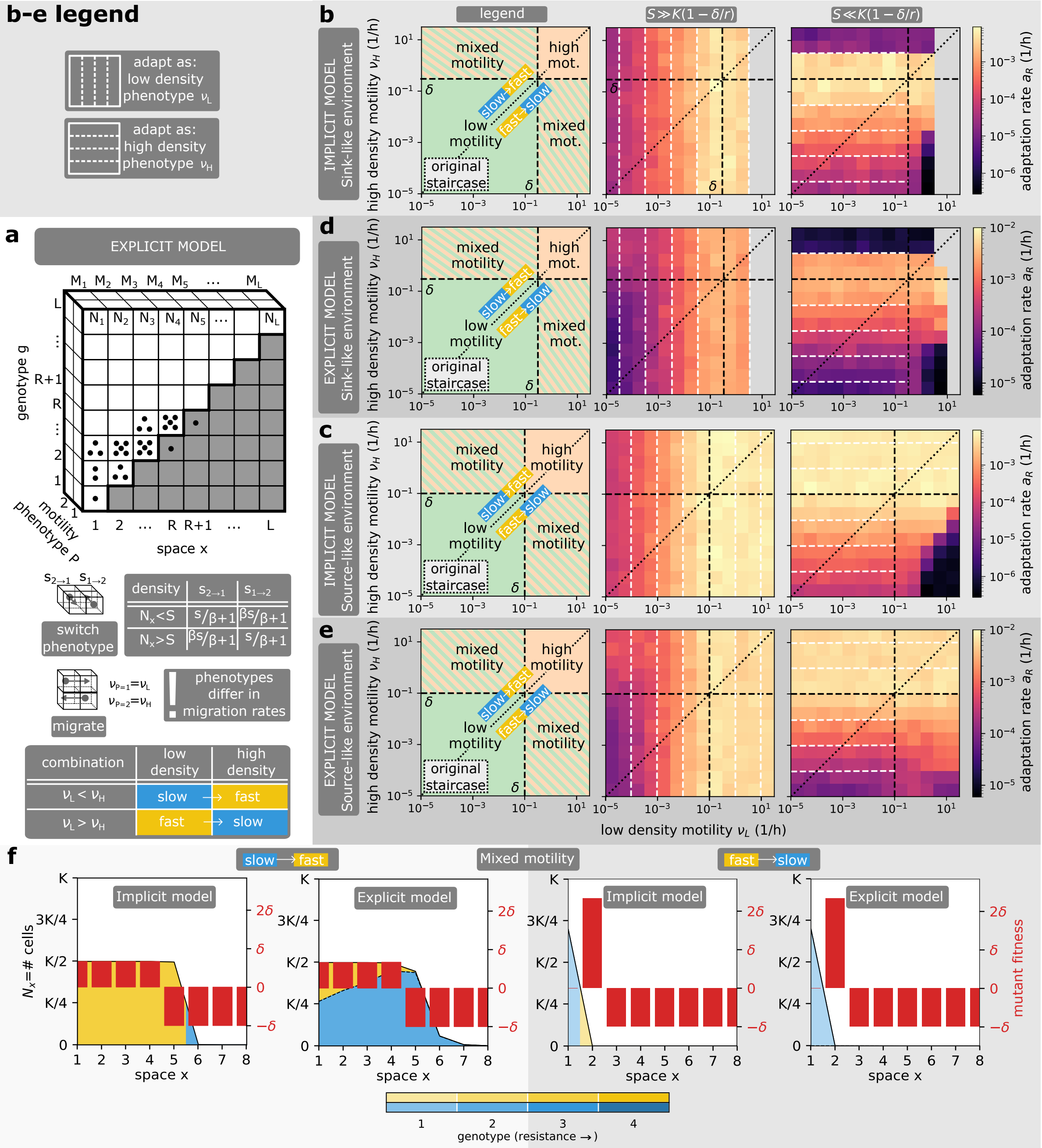


**Supplementary Figure** 8 | **Further effects of density-dependent motility. (a)** Explicit model. In the main text, we model motility phenotypes implicitly (implicit model). Alternatively, motility phenotypes can be modelled explicitly at each spatial position $x$ (explicit model). In this case, motility phenotypes are represented by a new dimension in the staircase model and are switched at a large rate ($s \gg \delta$) with a bias towards low-density (resp. high-density) phenotype at low (resp. high) density (characterised by $\beta \ll 1$), where low/high density is determined by comparing the density of cells $N_x$ at a spatial position $x$ to the switching threshold $S$. **(b-e)** Adaptation rates in source-like and sink-like environments of the implicit and explicit model. Adaptation rate is depicted as a heatmap on the $(\nu_L, \nu_H)$ plane for different switching thresholds $S$, where $\nu_{L,H}$ are the low-density/high-density motility rates. The $(\nu_L, \nu_H)$ plane can be partitioned into different combinations of adaptation regimes and its diagonal corresponds to a population of a single motility. The diagonal separates slow-to-fast and fast-to-slow switching combinations, which differ in relative motility at low-to-high density. Bacteria generically adapt as if all cells had the same effective motility, which corresponds to the intersection of the level sets (white dashed lines) with the diagonal. This effective motility generically matches the low-density (resp. high-density) motility at high (resp. low) threshold $S$. Exceptions to this rule for sink-like environments are explained in Fig. 4b (identical to panel **b** here), and we note that, in this case, the critical motility is slightly increased in the explicit model (panel **d**). In a source-like environment (panel **c**), the only exception to the rule of effective motility occurs at low $S$ in the mixed motility combination of the fast-to-slow switching case in the implicit model (panel **c**), where all cells in the overlap region switch to fast phenotype and are unlikely to start a growing mutant colony there. Moreover, the rule of effective motility does not have exceptions in the explicit model (panel **e**). **(f)** Wild-type profiles in the implicit and explicit model. The wild-type profiles are similar and differ only in predicted phenotypic proportions. The mutant fitness (red bars) is also similar in the implicit and explicit models, which further explains why these models exhibit the same adaptation dynamics. Find a detailed description of this model in Supplementary Note 6. Parameters: $L = 8$, $K = 10^5$, $r = 1/h$, $\delta = 0.1/h$ (source-like environment), $\delta = 0.3/h$ (sink-like environment), $\mu_b = 10^{-4}/h$, $\mu_f = 10^{-7}/h$.

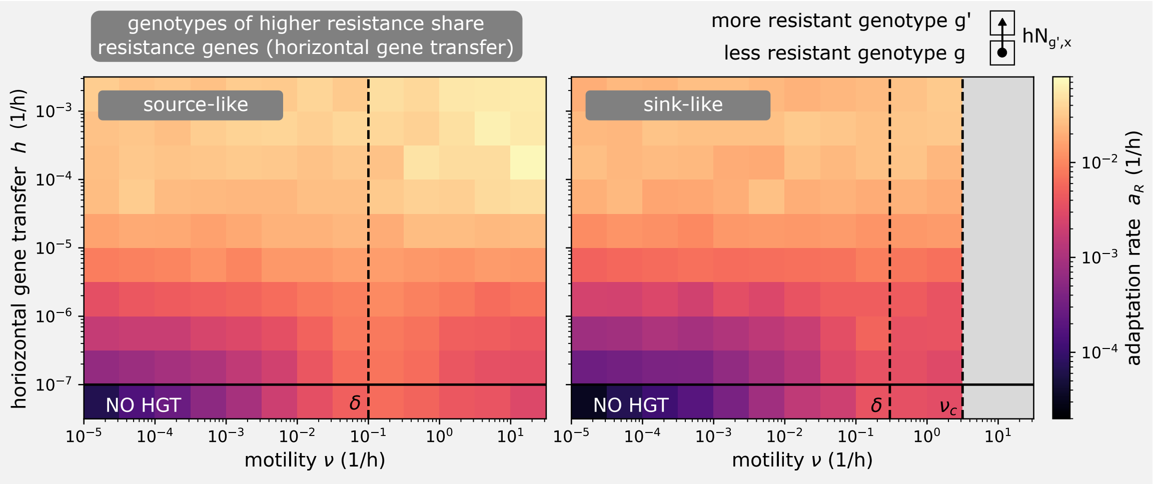


**Supplementary Figure** 9 | **Effect of horizontal gene transfer (HGT).** The heatmap of adaptation rate on the $(\nu, h)$ plane explains how the HGT rate $h$ changes the relationship between motility $\nu$ and adaptation rate $a_R$. HGT is modelled by allowing cells of less resistant genotypes $g$ to acquire resistance from $N_{x,g'}$ more resistant cells of genotype $g' > g$ located at the same position $x$ at a rate $h \times N_{x,g'}$. When HGT rate is low, the relationship between adaptation rate and motility is not affected: in the low motility regime, bacterial adaptation increases with motility but it decreases with motility in the high motility regime. When HGT rate is high, the adaptation rate is significantly increased and changes in motility do not play a significant role. However, the required HGT threshold $h \approx 10^{-5}$ corresponds to $hK/\delta \approx 1$ horizontal gene transfers per generation. Such HGT rates are considered very high, compared to the upper bound of $10^{-4}$ transfers per generation known from the literature.[S15] Moreover, HGT cannot influence the deadly motility regime (grey) as the donors of resistance genes do not have enough time to form before the wild-type goes extinct. A detailed description of this model can be found in Supplementary Note 4. Parameters: $L = 8$, $K = 10^5$, $r = 1/h$, $\delta = 0.1/h$ (left), $\delta = 0.3/h$ (right), $\mu_b = 10^{-4}/h$, $\mu_f = 10^{-7}/h$.

## REFERENCES


[S1] Bulmer, M. Multiple niche polymorphism. *The American Naturalist* **106**, 254–257 (1972).

[S2] Holt, R. D. & Gomulkiewicz, R. How does immigration influence local adaptation? a reexamination of a familiar paradigm. *The American Naturalist* **149**, 563–572 (1997).

[S3] Lenormand, T. Gene flow and the limits to natural selection. *Trends in Ecology & Evolution* **17**, 183–189 (2002).

[S4] Hastings, A. Dynamics of a single species in a spatially varying environment: the stabilizing role of high dispersal rates. *Journal of mathematical biology* **16**, 49–55 (1982).

[S5] Lipsitch, M. The rise and fall of antimicrobial resistance. *Trends in microbiology* **9**, 438–444 (2001).

[S6] Andersson, D. I. The biological cost of mutational antibiotic resistance: any practical conclusions? *Current opinion in microbiology* **9**, 461–465 (2006).

[S7] Hermsen, R., Deris, J. & Hwa, T. On the rapidity of antibiotic resistance evolution facilitated by a concentration gradient. *Proceedings of the National Academy of Sciences* **109**, 10775 – 10780 (2012).

[S8] Hermsen, R. & Hwa, T. Sources and sinks: a stochastic model of evolution in heterogeneous environments. *Physical review letters* **105**, 248104 (2010).

[S9] Köhler, T., Michea-Hamzehpour, M., Plesiat, P., Kahr, A.-L. & Pechere, J.-C. Differential selection of multidrug efflux systems by quinolones in pseudomonas aeruginosa. *Antimicrobial agents and chemotherapy* **41**, 2540–2543 (1997).

[S10] Sharma, S. K. & Mohan, A. Multidrug-resistant tuberculosis: a menace that threatens to destabilize tuberculosis control. *Chest* **130**, 261–272 (2006).

[S11] Kohanski, M. A., DePristo, M. A. & Collins, J. J. Sublethal antibiotic treatment leads to multidrug resistance via radical-induced mutagenesis. *Molecular cell* **37**, 311–320 (2010).

[S12] Oliveira, N. M. *et al.* Suicidal chemotaxis in bacteria. *Nature Communications* **13**, 7608 (2022).

[S13] Bru, J.-L. *et al.* Pqs produced by the pseudomonas aeruginosa stress response repels swarms away from bacteriophage and antibiotics. *Journal of bacteriology* **201**, e00383–19 (2019).

[S14] Von Wintersdorff, C. J. *et al.* Dissemination of antimicrobial resistance in microbial ecosystems through horizontal gene transfer. *Frontiers in microbiology* 173 (2016).

[S15] Niehus, R., Mitri, S., Fletcher, A. G. & Foster, K. R. Migration and horizontal gene transfer divide microbial genomes into multiple niches. *Nature communications* **6**, 1–9 (2015).

[S16] Cohan, F. M. The effects of rare but promiscuous genetic exchange on evolutionary divergence in prokaryotes. *The American Naturalist* **143**, 965–986 (1994).

[S17] Cohan, F. M. Does recombination constrain neutral divergence among bacterial taxa? *Evolution* **49**, 164–175 (1995).

[S18] Shapiro, B. J., David, L. A., Friedman, J. & Alm, E. J. Looking for darwin's footprints in the microbial world. *Trends in microbiology* **17**, 196–204 (2009).

[S19] Gevers, D. *et al.* Re-evaluating prokaryotic species. *Nature Reviews Microbiology* **3**, 733–739 (2005).

[S20] Levin, B. R. Periodic selection, infectious gene exchange and the genetic structure of e. coli populations. *Genetics* **99**, 1–23 (1981).

[S21] Baym, M. *et al.* Spatiotemporal microbial evolution on antibiotic landscapes. *Science* **353**, 1147–1151 (2016).

[S22] Zhang, Q. *et al.* Acceleration of emergence of bacterial antibiotic resistance in connected microenvironments. *Science* **333**, 1764–1767 (2011).

[S23] Liu, Z. & Papadopoulos, K. D. Unidirectional motility of escherichia coli in restrictive capillaries. *Applied and environmental microbiology* **61**, 3567–3572 (1995).

[S24] Kinosita, Y. *et al.* Distinct chemotactic behavior in the original escherichia coli k-12 depending on forward-and-backward swimming, not on run-tumble movements. *Scientific Reports* **10**, 15887 (2020).

[S25] Michelsen, O., Teixeira de Mattos, M. J., Jensen, P. R. & Hansen, F. G. Precise determinations of c and d periods by flow cytometry in escherichia coli k-12 and b/r. *Microbiology* **149**, 1001–1010 (2003).

[S26] Sloan, J. B. & Urban, J. E. Growth response of escherichia coli to nutritional shift-up: immediate division stimulation in slow-growing cells. *Journal of Bacteriology* **128**, 302–308 (1976).

[S27] Hastings, A. Can spatial variation alone lead to selection for dispersal? *Theoretical Population Biology* **24**, 244–251 (1983).

[S28] Barrett, P., Hunter, J., Miller, J. T., Hsu, J.-C. & Greenfield, P. matplotlib–a portable python plotting package. *Astronomical data analysis software and systems XIV* **347**, 91 (2005).
