# Supplementary material for: Bacterial motility can govern the dynamics of antibiotic resistance evolution
Source: Nat Commun. 2023 Sep 11;14:5584. doi: 10.1038/s41467-023-41196-8 (PMC10495427; doi:10.1038/s41467-023-41196-8)
Supplement: Supplementary file 3 — Description of Additional Supplementary Files [file 41467_2023_41196_MOESM3_ESM.pdf]

DESCRIPTION OF ADDITIONAL SUPPLEMENTARY FILES.

File Name: Supplementary Movie 1
Description:
Bacterial adaptation in the low and high motility regimes ($\nu < \delta$). Top: Low motility regime. Simulation of a bacterial population evolving antibiotic resistance in the staircase model in the low motility regime. It adapts and expands its range in a stepwise fashion, leaving behind an inclined comet tail (left) where shades of gray represent cell density in each compartment, which corresponds to a diversity of strains with different antibiotic susceptibility (right). Bottom: High motility regime. Note that the comet tail is now horizontal (left), and the population is made of a single strain that can grow in $x \leq R$ (right). Find a full description in the caption of Fig. 1. Parameters used: $L = 8$, $K = 10^5$, $r = 1h^{-1}$, $\delta = 0.1h^{-1}$, $\mu_f = 10^{-7}h^{-1}$, $\mu_b = 10^{-4}h^{-1}$, with $\nu = 0.01h^{-1}$ for the low motility regime and $\nu = 1h^{-1}$ for the high motility regime.

File Name: Supplementary Movie 2
Description:
Effect of chemotaxis. Similar to high motility in Supplementary Movie 1 but now $p > 0.5$ for positive chemotaxis (top) and $p < 0.5$ for negative chemotaxis (bottom). Bacterial chemotaxis determines the shape of the population profile. While positive chemotaxis increases the density of cells in high antibiotic concentrations, negative chemotaxis increases the density of cells in low antibiotic concentrations. In particular, these population densities are very high and can be larger than the carrying capacity, as chemotaxis continuously imports cells from neighbouring compartments. Such unbalanced population distribution reduces the adaptation rate of bacteria (Supplementary Fig. 6). Parameters used: $L = 8$, $K = 10^5$, $r = 1h^{-1}$, $\delta = 0.1h^{-1}$, $\mu_f = 10^{-7}h^{-1}$, $\mu_b = 10^{-4}h^{-1}$, $\nu = 1h^{-1}$, with $p = 0.6$ for positive chemotaxis and $p = 0.2$ for negative chemotaxis.

File Name: Supplementary Movie 3
Description:
Effect of switching rate in stochastic phenotypic switching. Simulation of a bacterial population evolving antibiotic resistance in the staircase model with stochastic switching between motility phenotypes for the mixed motility combination $\min(\nu_1, \nu_2) < \delta < (\nu_1 + \nu_2)/2$ when the switching rate is low (top) and high (bottom). For both switching rates, we show the slow subpopulation in blue, and the faster subpopulation in yellow. Find a full description in the caption of Fig. 3. Parameters used: $L = 8$, $K = 10^5$, $r = 1h^{-1}$, $\delta = 0.1h^{-1}$, $\mu_f = 10^{-7}h^{-1}$, $\mu_b = 10^{-4}h^{-1}$, $\nu_1 = 10^{-2}h^{-1}$, $\nu_2 = 2h^{-1}$, with $s = 10^{-3}h^{-1}$ for low switching rate and $s = 5h^{-1}$ for high switching rate.

File Name: Supplementary Movie 4
Description:
Effect of density-dependent motility. Simulation of a bacterial population evolving antibiotic in the staircase model with slow-to-fast density-dependent motility, where slow/fast signifies the relative magnitude of motility at low/high population density. If the environment is source-like (left), the faster high-density population bulk (yellow) expands its range by pulling the slower low-density population front (blue). If the environment is sink-like (right), and the faster high-density motility phenotype moves above the critical motility, this expansion is halted. As a result of this reduced antibiotic exposure, the adaptation rate is decreased in the top row of Supplementary Fig. 8b (sink-like environment) but not in the top row of Supplementary Fig. 8c (source-like environment). Parameters used: $L = 8$, $K = 10^5$, $r = 1h^{-1}$, $\delta = 0.1h^{-1}$ (left) and $\delta = 0.3h^{-1}$ (right), $\mu_f = 10^{-7}h^{-1}$, $\mu_b = 10^{-4}h^{-1}$, $\nu_L = 10^{-3}h^{-1}$, $\nu_H = 10^{0.5}h^{-1}$, $S = 10^4$.

File Name: Supplementary Movie 5
Description:
Comparison of models in the deadly motility regime. Simulation of bacterial populations that display the deadly motility regime in the original model with simple unbiased motility (upper row), the model with stochastic switching motility (middle row) and the model with density-dependent motility (lower row). In all models, bacteria have the same effective motility, which is higher than the critical motility. Bacterial populations go extinct in all models on the same timescale. Parameters used: $L = 8$, $K = 10^5$, $r = 1h^{-1}$, $\delta = 0.3h^{-1}$, $\mu_f = 10^{-7}h^{-1}$, $\mu_b = 10^{-4}h^{-1}$. In the original model $\nu = 20h^{-1}$; in the stochastic switching model $\nu_1 = 19.999h^{-1}$, $\nu_2 = 0.001h^{-1}$, $s = 100$; in the density-dependent motility model $\nu_L = 20h^{-1}$, $\nu_H = 10^{-3}h^{-1}$, $S = 6 \times 10^4$.
